# Demystifying the persistent pneumothorax: role of imaging

**DOI:** 10.1007/s13244-016-0486-5

**Published:** 2016-04-21

**Authors:** Apeksha Chaturvedi, Steven Lee, Nina Klionsky, Abhishek Chaturvedi

**Affiliations:** Department of Imaging Science, University of Rochester Medical Center, P.O. Box no. 648, 601 Elmwood Ave, Rochester, NY 14642 USA

**Keywords:** Persistent pneumothorax, Secondary pneumothorax, Bronchopleural fistula, CT, Radiography

## Abstract

Evaluation for pneumothorax is an important indication for obtaining chest radiographs in patients who have had trauma, recent cardiothoracic surgery or are on ventilator support. By definition, a persistent pneumothorax constitutes ongoing bubbling of air from an in situ chest drain, 48 h after its insertion. Persistent pneumothorax remains a diagnostic dilemma and identification of potentially treatable aetiologies is important. These may be chest tube related (kinks or malposition), lung parenchymal disease, bronchopleural fistula, or rarely, oesophageal-pleural fistula. Although radiographs remain the mainstay for diagnosis and follow up of pneumothorax, computed tomography (CT) is increasingly being used for problem solving. Aetiology of persistent air leak determines the optimal treatment. For some, a simple repositioning of the chest tube/drain may suffice; others may require surgery. In this pictorial review, we will briefly describe the physiology of pneumothorax, discuss imaging features of identifiable causes for persistent pneumothorax and provide a brief overview of treatment options. Specific aetiology of a persistent air leak may often not be immediately discernible, and will need to be carefully sought. Accurate interpretation of imaging studies can expedite diagnosis and facilitate prompt treatment.

*Key points*

• *Persistent pneumothorax is defined as a leak persisting for more than 2 days*.

• *Radiographs can identify chest-tube-related causes of pneumothorax*.

• *CT is the most useful test to identify other causes*.

• *Penetrating thoracic injury can cause fistulous communication resulting in a persistent pneumothorax*.

• *Discontinuity of visceral pleura identified by CT may indicate a bronchopleural fistula*.

## Introduction

Pleural space is a potential space normally filled with few millilitres of fluid with a negative intrapleural pressure [1]. Pneumothorax is defined as an abnormal accumulation of gas within the pleural space [2]. Pneumothoraces can be classified as spontaneous, post traumatic or iatrogenic [3]. A spontaneous pneumothorax occurs in the absence of a triggering event and can be subclassified as primary or secondary, depending on whether or not there is associated disease such as chronic obstructive pulmonary disease (COPD) [4]. Post-traumatic pneumothorax can occur as a consequence of blunt or penetrating trauma to the chest, whereas iatrogenic pneumothorax occurs as a complication of a diagnostic or therapeutic procedure. Incidence of pneumothorax is higher in cigarette [5] and cannabis smokers.

**Fig. 1:**
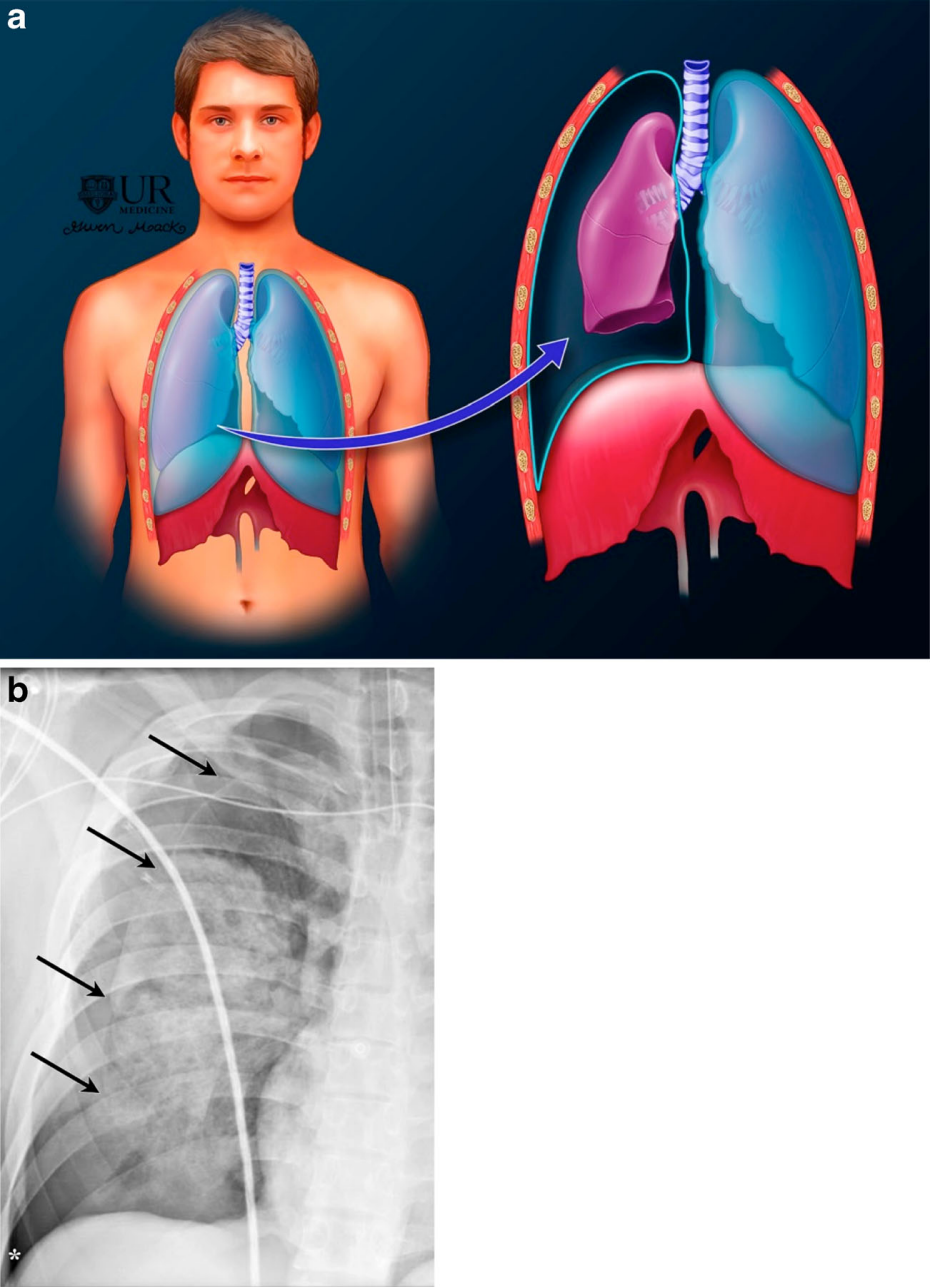
Pneumothorax depicted on illustration (**a**) and frontal chest radiograph (**b**). The visceral pleural line (marked by *black arrows* on b) is displaced medially and a lucency (air) intervenes between the chest wall and the outer surface of the right lung. Right lung is partially collapsed. * indicates the deep sulcus sign

Tension pneumothorax is identified as a distinct entity and characterized by progressive build up of air within the pleural space from a one-way valve (either due to chest wall or pulmonary injury), which allows air to enter the pleural space, but not to escape (Fig. 1b). Pneumothorax ex vacuo is a distinct entity seen with lobar atelectasis from acute bronchial obstruction. The resultant increased negative intrapleural pressure draws gas into the pleural space [6]. This type of pneumothorax spontaneously resolves once the bronchial obstruction is relieved [6, 7]. This term has recently also been used to refer to the development of gas in the pleural space because the lung is unable to expand and fill the thoracic cavity after evacuation of pleural fluid [8].

## Pneumothorax and physiology of respiration

Lungs float in the thoracic cavity surrounded by pleural fluid in the pleural space. Pleural space is a potential space containing a few millilitres of fluid [9]. Intrapleural pressures are normally a slight suction or slightly negative; generally about−5 cm H_2_O. During normal inspiration, outward expansion of lungs results in further drop in intrapleural pressure (to about−7.5 cm H_2_O), thereby driving inflow of atmospheric air into the alveoli. During expiration, these events are reversed.

If air enters the pleural space, as in a pneumothorax, normal negative pressure within the pleural space is disrupted, thus interrupting normal dynamics of airflow. To exemplify, a change in transpleural pressure from−5 to−2.5 cm H_2_O results in a 33 % decrease in vital capacity by compressing the lung and altering thoracic wall recoil [10].

## Grading of air Leak

Air leaks can be classified into four categories based on clinical findings [11]:

1. Forced expiratory – air leak present only with cough. This is the most common type of air leak after elective pulmonary surgery.
2. Expiratory – air leak only present on expiration. This is commonly seen in patients with alveolopleural fistula (APF).
3. Inspiratory – air leak only present on inspiration. This is seen in patients receiving mechanical ventilation, or with sizable APF or a small bronchopleural fistula (BPF)
4. Continuous – air leak present during the entire respiratory cycle. This is the least common type and is seen in patients on mechanical ventilation or with BPF.

## Imaging

### Radiographs

Chest radiographs are the cornerstone for diagnosing pneumothorax. If a pneumothorax is present, a white visceral pleural line separates the lung from the chest wall, with loss of normal lung markings peripheral to this white line (Fig. 1a, b). Occasionally, the lung on the affected side may completely collapse.

Erect inspiratory posterior-anterior radiographs are generally preferred for assessment of pneumothorax. In critically ill patients, anterior-posterior, supine or semi-erect radiographs may be obtained. In difficult cases, lateral decubitus radiographs can help identify an anterior pneumothorax.

Identification of a pneumothorax on portable radiographs can be challenging, and presence of a “deep sulcus” sign constitutes an important clue (Fig. 1b). Expiratory radiographs are no longer recommended [12]. Note that the most nondependent portion of the pleural space is the inferior lateral hemithorax, especially in children. Therefore, it is important that the lateral costophrenic angles be included on all supine radiographs being used to evaluate for pneumothorax.

### Ultrasound

Ultrasound has developed as a robust tool for identification, as well as follow-up, of pneumothorax. In normal subjects, respiration dependent movement of visceral pleura on parietal pleura can be seen by ultrasound. This sliding movement or lung pulse at the pleural interface indicates that there is no pneumothorax at the site of the examination [8]. Air in the pleural space prevents visualization of visceral pleural movement [13]. Therefore, in the presence of pneumothorax, the gliding/sliding sign and comet-tail artefacts disappear. A completely motionless pleural line using real-time ultrasound is called as the “stratosphere” sign [14].

The role of ultrasound towards identifying the aetiology of a persistent pneumothorax is however, not well studied; therefore, for this purpose, ultrasound cannot be recommended at present.

### CT

Computed tomography (CT) is the most sensitive and specific test for diagnosis of pneumothorax and is now considered the standard of reference. It can be performed expeditiously and is emerging modality of choice to identify a persistent air leak. Multiplanar reconstruction (MPR), maximum intensity projections (MIP), minimum intensity projection (minIP), volume rendering and virtual bronchoscopy help to optimally demonstrate the defect as well as provide a road map for surgical intervention. In addition, thin slices and sharper image reconstruction algorithm may help identify the fistula not obvious on thicker slices reconstructed with smoother algorithm.

CT is particularly useful in identifying secondary causes of pneumothorax or persistent air leaks. It helps distinguish a bulla from a loculated pneumothorax due to pleural adhesions and malposition of chest tubes. When evaluating secondary causes of persistent pneumothorax, use of intravenous contrast helps identify vasculature and empyema. Endobronchial valves, originally designed for bronchoscopic lung volume reduction, have been used under a humanitarian use exception for the treatment of bronchopleural fistula [15]. Careful review of the CT can help in identifying the bronchopulmonary segment from which the air leak is occurring. In patients with suspected oesophagorespiratory fistula, careful use of positive oral contrast may help in defining the leak if it is not visible on noncontrast CT. Minimal amount of contrast should be used with care taken to avoid aspiration. Indeed, a CT obtained immediately after contrast oesophagogram increases the accuracy of diagnosing oesophageal perforation [16]. The ingestion of water-soluble contrast prior to CT may display the site of extravasation [17].

In our practice, when an oesophagorespiratory fistula is suspected, the following protocol is followed:

Initial non-contrast CT is obtained to identify any CT signs suggestive of perforation/fistula, such as: paraoesophageal extra luminal air, fluid collections in the mediastinum or pleura, oesophageal laceration [18]. If a definitive diagnosis of a fistulous communication can be made, no further imaging is obtained and the patient proceeds for therapeutic endoscopy/bronchoscopy. If the initial findings from the noncontrast CT are equivocal or the only findings are oesophageal wall thickening or fluid in mediastinum/pleura with no clear evidence of a fistula, a contrast enhanced CT is obtained with diluted water soluble oral and intravenous (IV) contrast (Table 1), as has been previously described [19]. In our practice, we limit the use of effervescent granules to only those patients who have a gastric interposition graft after oesophagectomy.

**Table 1:** Protocol for obtaining CT in patients with a suspected oesophageal respiratory fistula

	Z axis coverage	IV Contrast	Oral contrast*
Initial noncontrast	Thoracic inlet – below diaphragm	None	None
Second phase with IV and oral contrast	Thoracic inlet – below diaphragm	50–75 cc at 2–3 cc/s, images acquired at 40 sec delay	75–300 mL of an aqueous solution consisting of IV iodinated contrast material Omnipaque 350
Prone/Decubitus (if needed)	Limited over region of suspicious perforation	None	50 mL of an aqueous solution consisting of IV iodinated contrast material Omnipaque 350

^*^ At least 1 mL of Omnipaque 350 per 37.4 cc of water [60]. A more concentrated 10 % solution is better to delineate these defects [19]. If patient is able to swallow, they are instructed to hold their breath and swallow; otherwise, they are asked to sip continuously from a cup with a straw. In unconscious patients, contrast may be injected thorough a nasogastric (NG) tube

### Quantifying pneumothorax

**Fig. 2:**
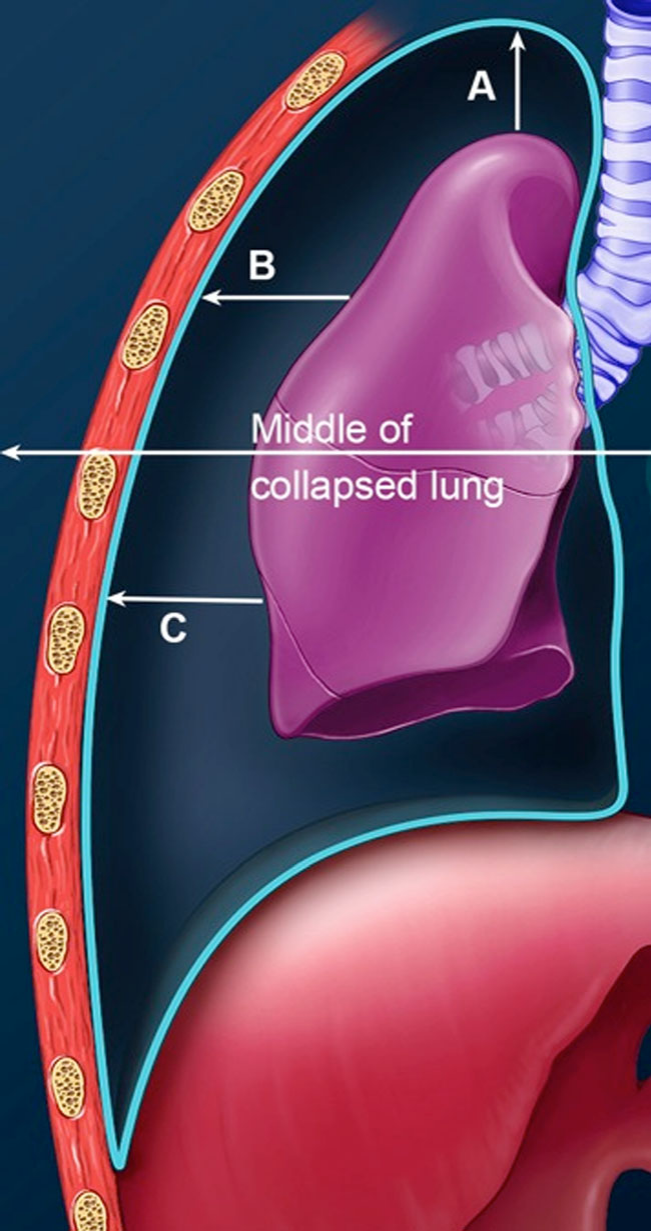
Volumetric assessment of pneumothorax on a frontal chest radiograph. This is performed using the formula: pneumothorax % = 4.2 + [4.7 (A + B + C)]. ‘A’ represents the distance from the lung to the cupola. ‘B’ represents the distance from the upper mid collapsed lung to the chest wall and ‘C’ represents the distance from the lower mid collapsed lung to the chest wall. If this number is greater than 25 %, chest tube drainage is recommended

Different guidelines have been used to quantify pneumothorax. The British Thoracic Society (BTS) guidelines divide pneumothoraces into small and large based on the distance from visceral pleural surface (lung edge) to chest wall at the level of the hilum, with less than 2 cm being small and more than 2 cm being considered as large and corresponding to 50 % of the hemithorax being occupied by pleural air [2]. The volume of pneumothorax can also be calculated from an erect posterioranterior (PA) chest radiograph using this formula (Fig. 2): pneumothorax % = 4.2 + [4.7 (A + B + C)], where A, B, and C represent the intrapleural distances measured at the apex, hila and lower half of the collapsed lung [20]. This method includes also the regression analysis based on volume measurements from helical CT.

### Persistent pneumothorax/air leak

The definition of a persistent air leak varies from study to study. In postoperative studies, a pneumothorax persisting beyond the first week is considered a persistent air leak [21]. The BTS defined it as the continued bubbling of air through a chest drain after 48 h in situ [2].

There are differences in the management guidelines proposed by the American College of Chest Physicians (ACCP) and the BTS. The BTS recommends a thoracic surgery consult if the air leaks persist beyond 2 days or if the lung does not re-expand, while the ACCP recommends intervention for air leaks persisting beyond 4 days in primary spontaneous pneumothorax and over 5 days in secondary pneumothorax. For the purpose of this review, we will consider a persistent pneumothorax as an air leak persisting beyond 2 days. The likely causes of a persistent pneumothorax are presented in Table 2.

**Table 2:** Aetiology of persistent air leak/pneumothorax

Cause	Examples
Mimics	Skin fold Companion shadow Bulla Eloessar flap Ex vacuo
Chest Tube	Kink Obstruction Malposition Incomplete seal
Bronchopleural fistula (BPF)	Bronchial stump dehiscence Iatrogenic Traumatic Erosive
Alveolopleural fistula (APF)	Ruptured bulla Traumatic Necrotizing pneumonia Ulcerated Lung cancer Metastases: osteosarcoma Bronchopleural fistula
Others	Esophagopleural fistula

Some of these can cause either an APF or a BPF

## Mimics of pneumothorax

**Fig. 3:**
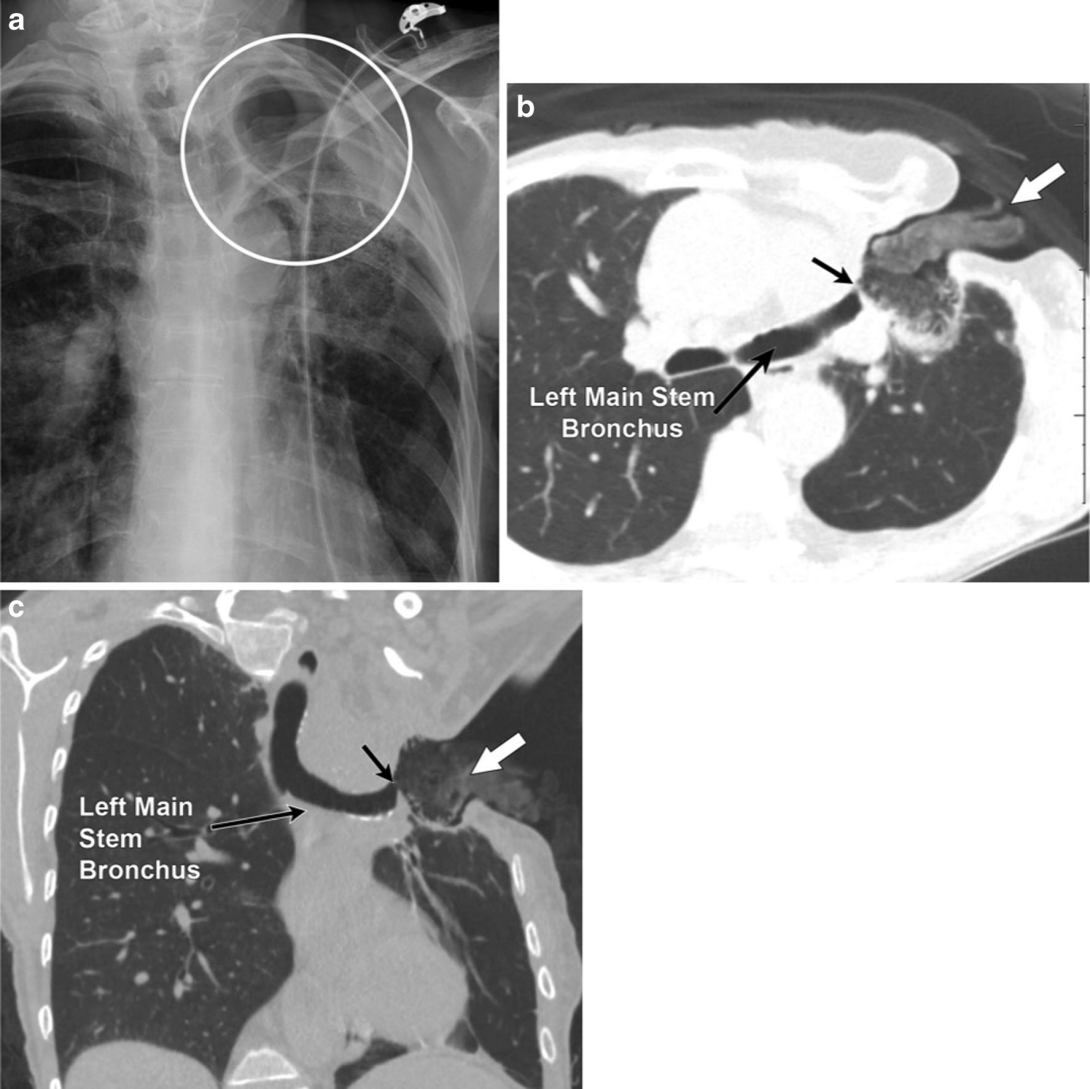
A 67-year-old female; status post Eloesser flap for left empyema. Frontal chest radiograph (**a**) demonstrates lucency over the left chest apex suggesting pneumothorax (marked by a *circle*). Axial (**b**) and coronal (**c**) CT images using lung windows demonstrate the Eloesser flap (*solid white arrow*). In addition, a bronchopleural fistula is also identified (*black arrow*)

**Fig. 4:**
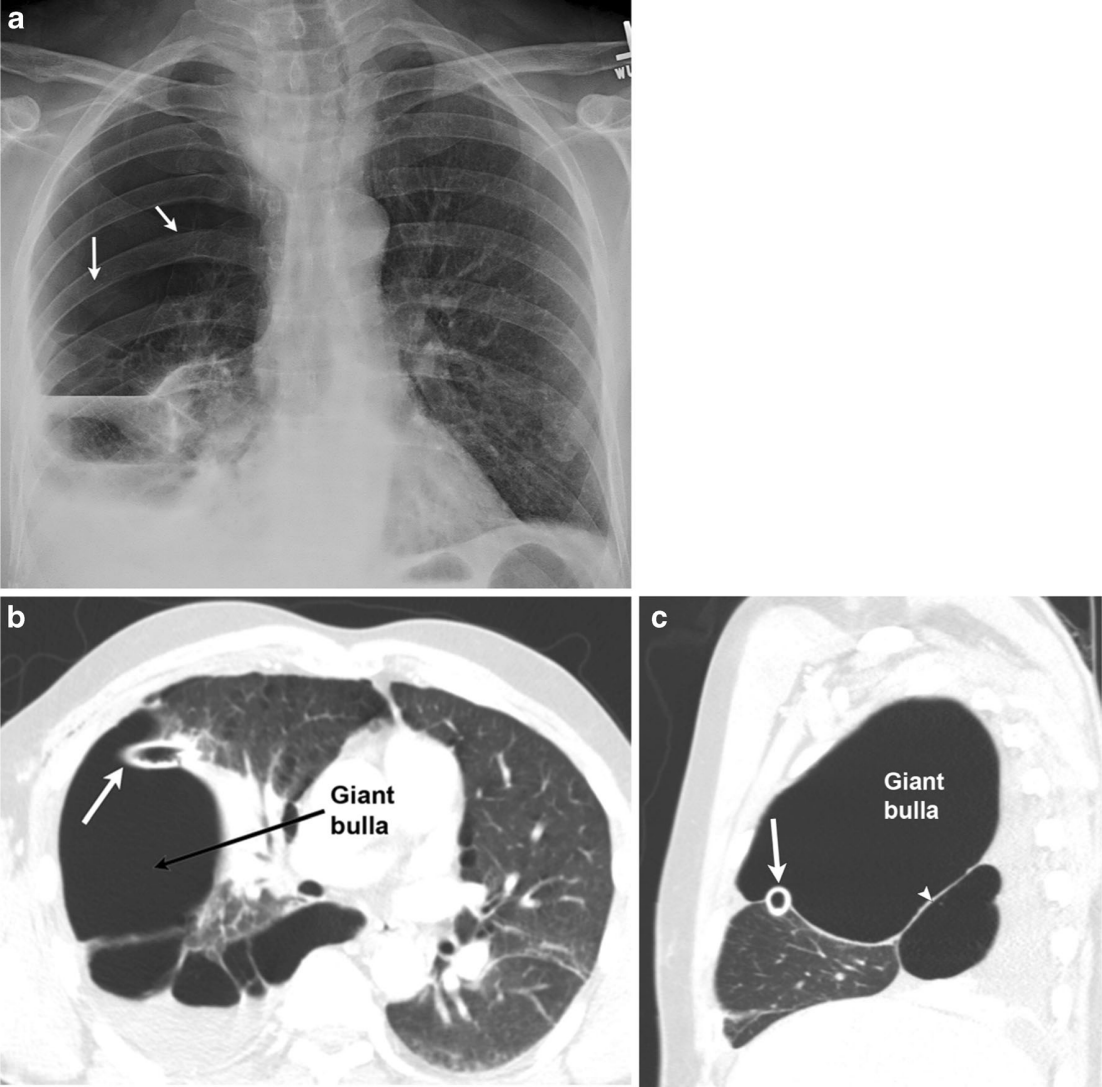
A 46-year-old smoker presenting to the emergency department (ED) with chest pain. Frontal chest radiograph (**a**) demonstrates hyperlucency through the right chest (*thin arrows*) with few dependent fluid levels; this was interpreted as a hydro pneumothorax. Post chest tube placement, both axial (**b**) and sagittal (**c**) CT images demonstrate chest tube within the right minor fissure (*solid white arrow*), abutting a giant bulla (defined as bulla occupying more than 30 % of the hemithorax). The bulla can be differentiated from a pneumothorax by the presence of septae (*arrowhead*) and compression of the lung parenchyma, unlike a pneumothorax where a visceral pleural line should be seen. The initial radiographic assessment was therefore inaccurate

While evaluating for pneumothorax, it is important to differentiate true pneumothorax and the mimickers. Important mimickers of pneumothorax on a radiograph include: skin folds, scapular margins and companion shadows along the inferior margins of the ribs [22]. These have been discussed extensively previously in the literature; hence, for the purpose of this review we will only discuss the Elosser flap. The Elosser flap (Fig. 3) was originally developed in 1935 by Leo Eloesser to treat tuberculous pleural space infections. It has since evolved to treat persistent pleural space infections associated with bronchopleural fistulas (BPF) as well as post-pneumonectomy BPF [23]. It involves creating a permanent skin-lined opening in the chest wall with infolding of the cutaneous skin flaps into the thorax. This permanent opening is created and prevents accumulation of pleural effusion, allowing lung re-expansion. In addition, a giant bulla can also mimic a pneumothorax (Fig. 4).

## Chest drains/tubes

Malposition of the chest tube is common, in particular in trauma patients where these may be inserted in suboptimal conditions [24]. This can lead to suboptimal drainage of the pleural fluid or pneumothorax.

**Fig. 5:**
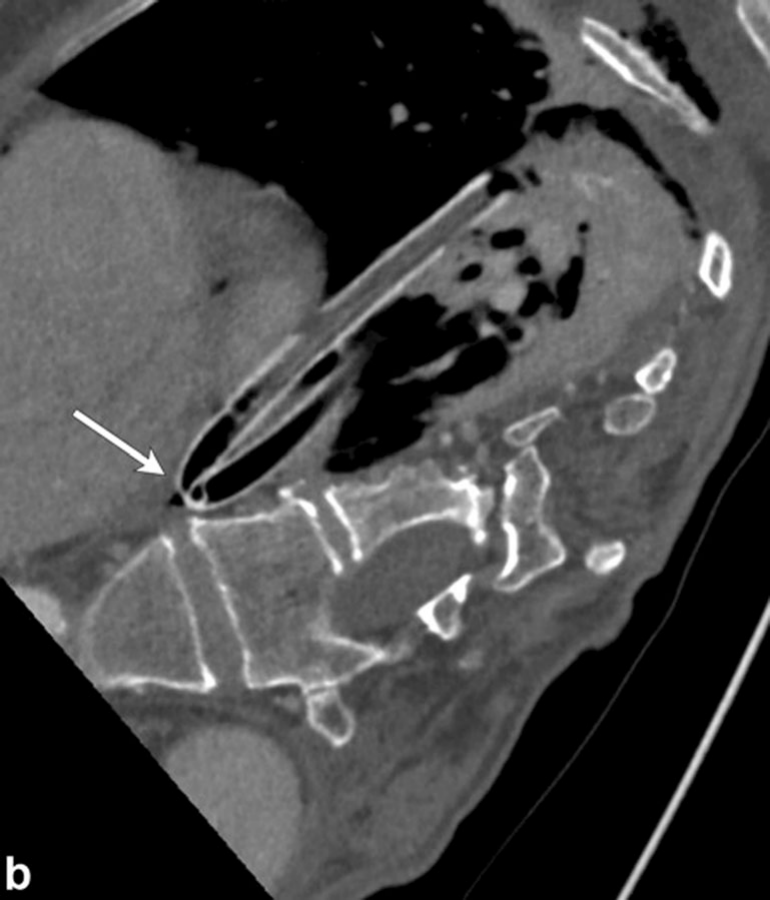
An 87-year-old female presenting with multiple rib fractures after falling down stairs. Frontal chest radiograph (**a**) and oblique multiplanar reconstruction from CT (**b**) demonstrate acute kink involving the chest tube (*arrow*); this was the etiology of a nonresolving pneumothorax

**Fig. 6:**
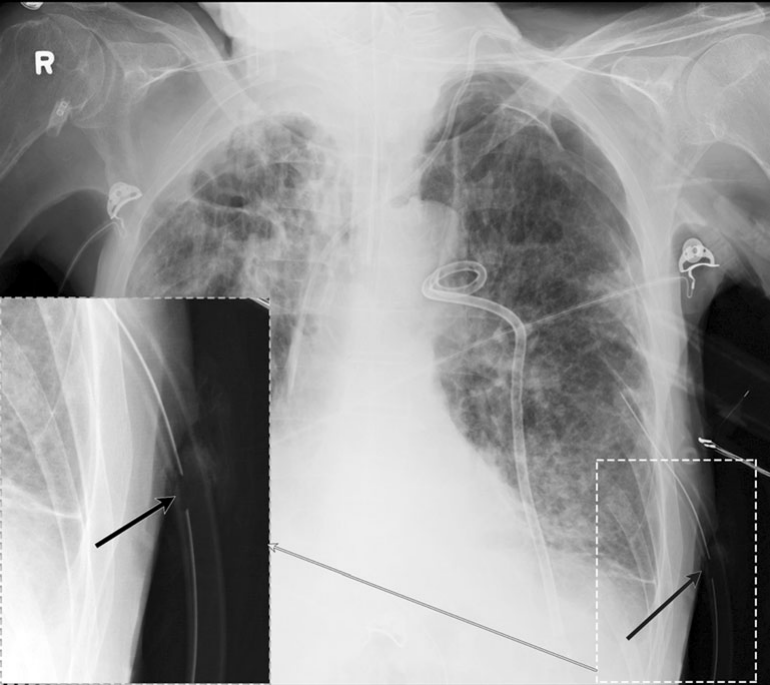
An 88-year-old male with underlying COPD and bullous emphysema presented with a spontaneous left pneumothorax. Despite subsequent chest tube and anterior pleural catheter placement, left pneumothorax persisted. Upon careful review of the radiograph, side hole of the left chest tube was outside the pleural cavity (*black arrow*) and communicated with the atmospheric air

A focal kink in the extra- or intrathoracic portion of the chest tube will obstruct the lumen and lead to suboptimal evacuation of the pneumothorax (Fig. 5). Incomplete insertion of the chest tube with its side hole outside the pleural cavity can lead to suboptimal evacuation of air (Fig. 6). If the side hole (sentinel eye) is outside the chest wall, it may lead to backflow of atmospheric air into the pleural space. Intrafissural position of the chest tube may or may not have clinical consequences [25, 26]. It can lead to delayed or poor evacuation of the pleural effusion or pneumothorax [27].

**Fig. 7:**
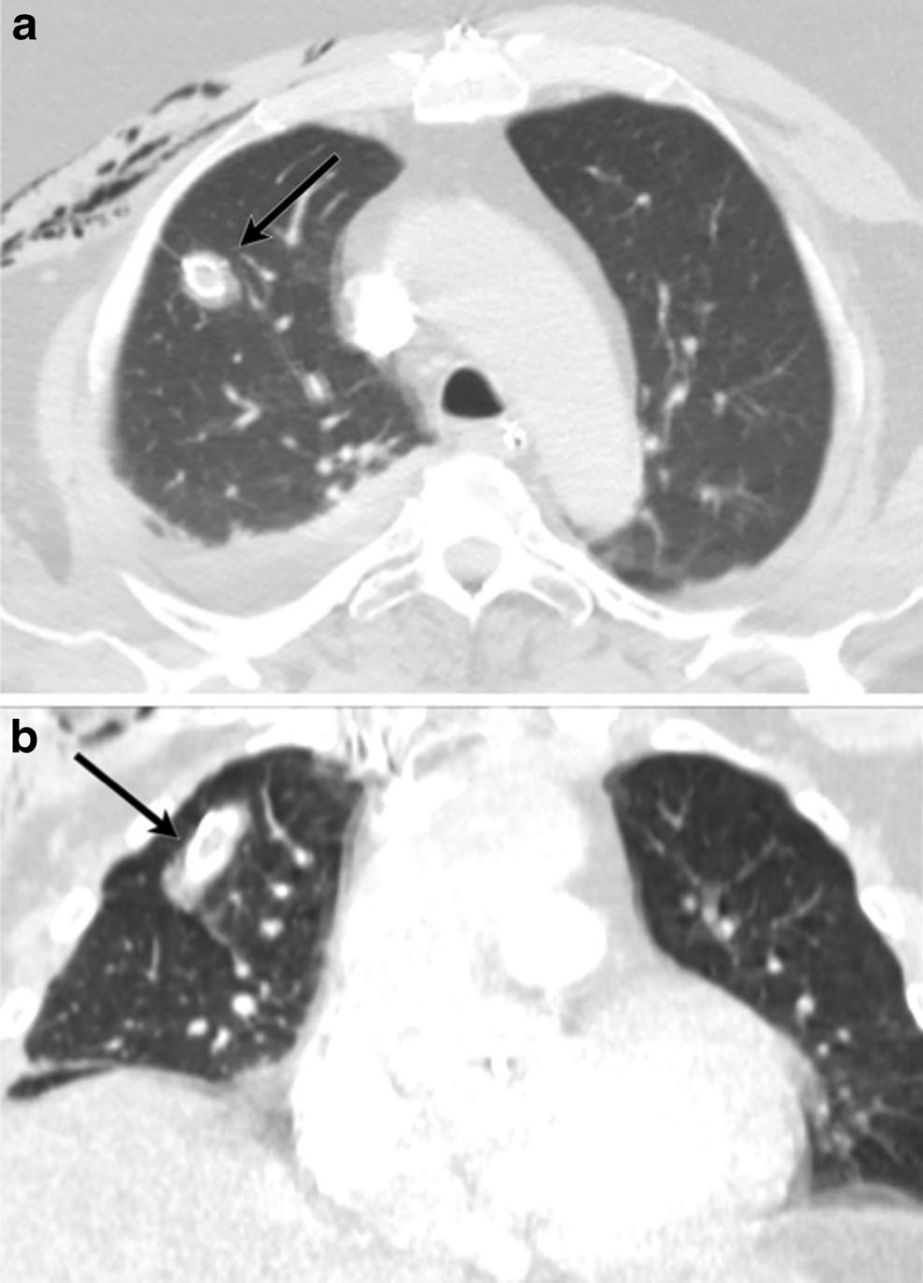
A 69-year-old male struck by a motor vehicle. CT was obtained to assess etiology of a non-resolving pneumothorax. Axial (**a**) and coronal (**b**) CT images through the chest demonstrate intra parenchymal placement of chest tube within the right upper lobe. Ground glass opacity (*arrow*) surrounding the chest drain represents lung laceration. Incidentally noted right pleural fluid and right anterior chest wall soft tissue emphysema

Intraparenchymal positioning of the chest tube can be due to underlying lung parenchymal diseases. Alternatively, pleural adhesions or inadvertent, too-vigorous insertion can place the chest tube within the lung parenchyma, causing lung contusion and/or laceration. The radiographs may be completely unremarkable or may demonstrate an opacity surrounding the intrathoracic portion of the chest tube, representing surrounding hematoma. CT with particular attention to coronal and sagittal images demonstrates the lung completely surrounding the tube (Fig. 7). Indeed, lung is the most commonly injured organ during chest tube placement. Parenchymal tube placement can result in persistent tubular opacities representing the healed tract, or may cause a bronchopleural fistula.

**Fig. 8:**
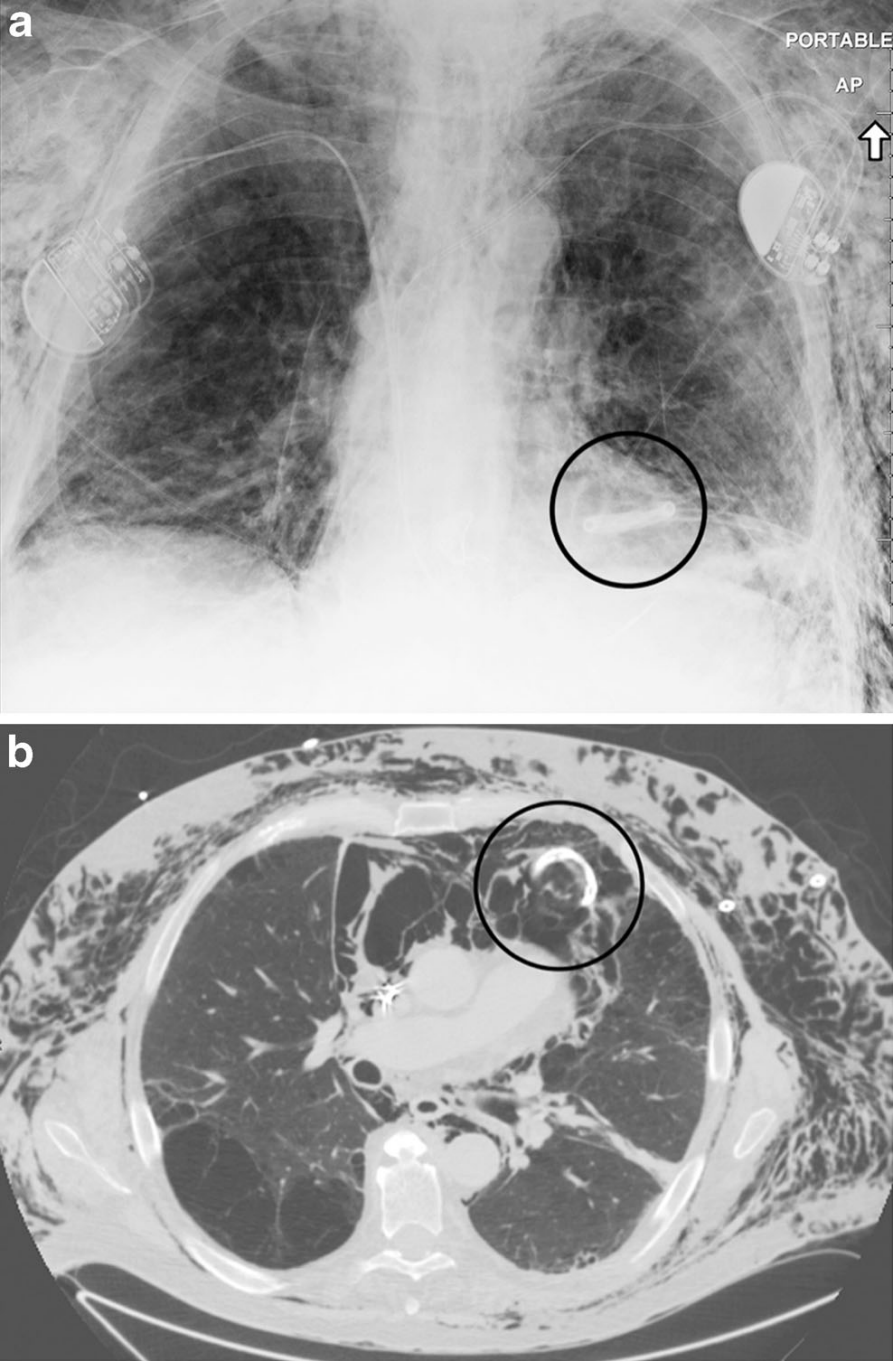
A 67-year-old male with multiple rib fractures status post fall. Chest radiograph (**a**) demonstrates a pleural pigtail drain projecting over the lower left chest (*circle*). Axial CT image through the upper thorax (**b**) demonstrates anterior mediastinal location of the drain (*circle*) with extensive pneumomediastinum and chest wall emphysema

**Fig. 9:**
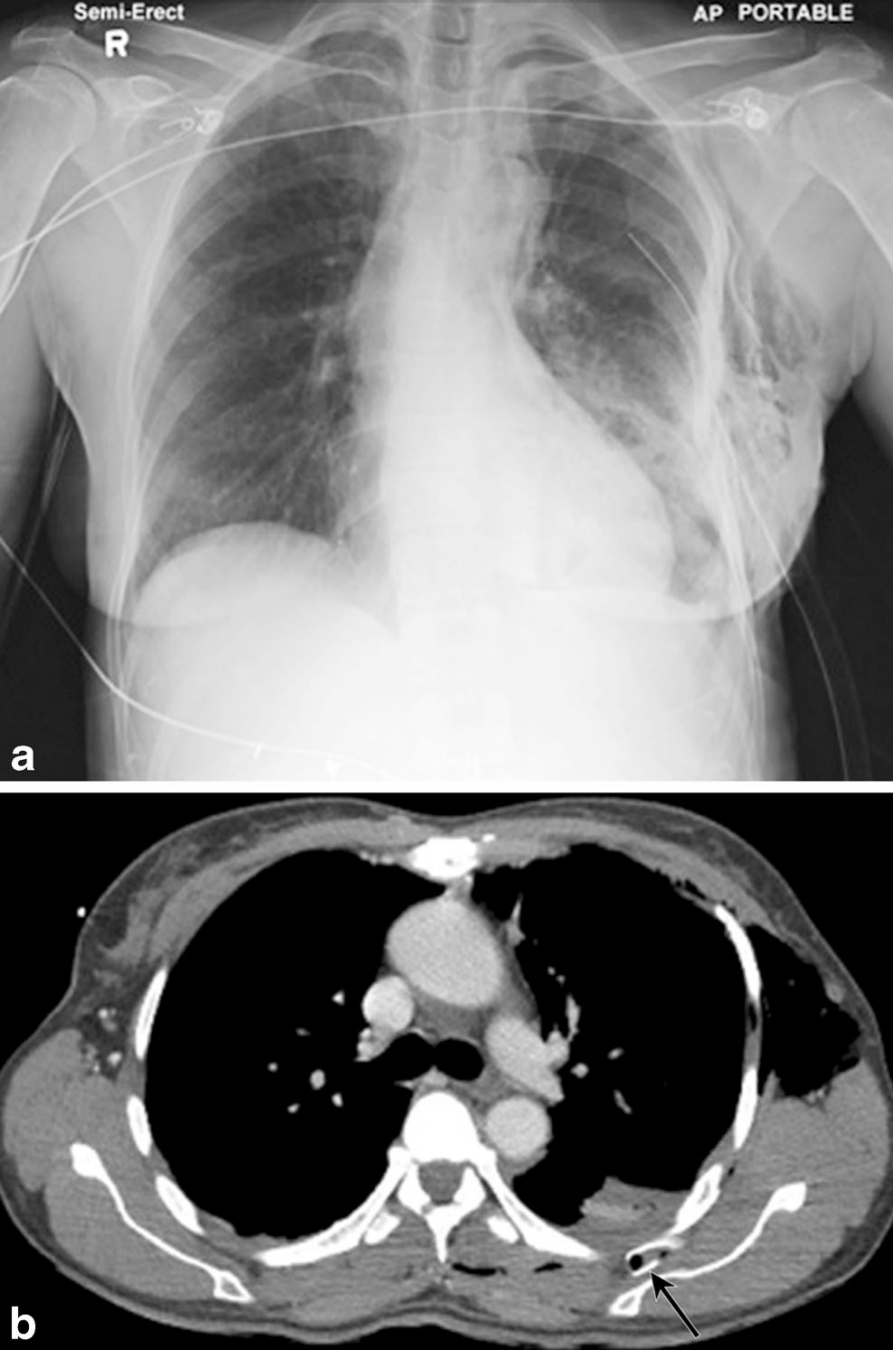
A 52-year-old female status post motor vehicle accident had an emergent chest tube placement by EMR. Semi-erect AP radiograph (**a**) demonstrates chest tube projecting over the left lateral upper-mid chest; positioning was thought appropriate. However, subsequently performed CT (**b**) demonstrates chest tube tip within the soft tissues of the posterior chest wall (*black arrow*), between the outer surface of the rib and scapula. Tube was outside the pleural cavity

A tube inserted too far can lead to mediastinal placement (Fig. 8). Complications of mediastinal tube placement include perforation of oesophagus, pulmonary artery and heart. Muscular chest wall, obesity or presence of chest wall emphysema can lead to the tube being placed in the chest wall outside the pleural cavity. When the tube is placed across the lateral chest wall, it can be recognized on radiographs; however, for anteriorly or posteriorly placed tubes or drains, such as for loculated pneumothorax (Fig. 9), CT is more useful.

**Fig. 10:**
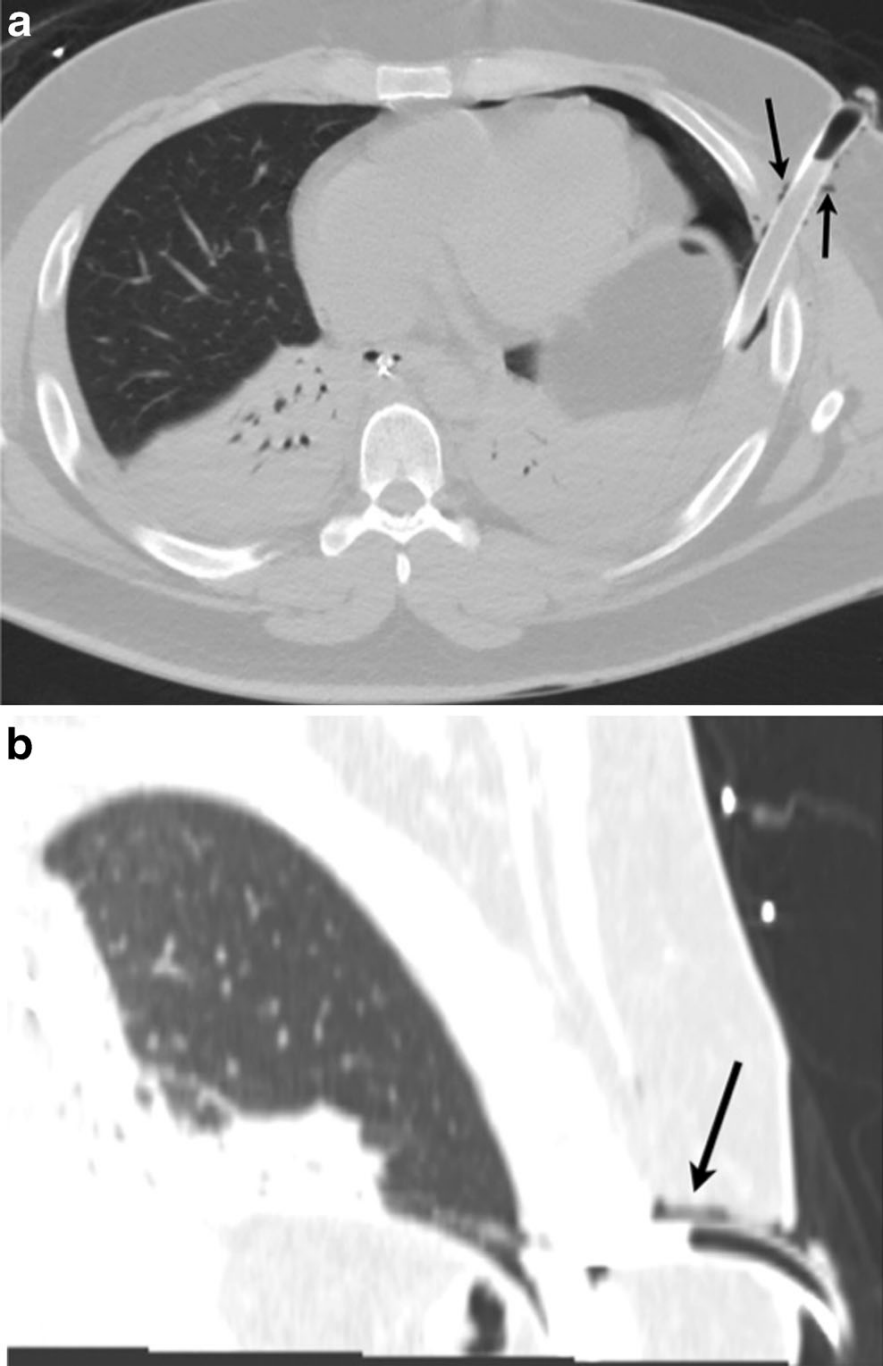
A 54-year- old-male status post aortic aneurysm repair presented with a persistent left pneumothorax. On axial (**a**) and coronal (**b**) CT images, air surrounds the portion of the chest tube coursing through the chest wall (*arrows*). This indicates an incomplete seal. If the site of thoracotomy is not optimally occluded with surgical dressing, or if the incision is too large relative to the tube, an air leak may develop. This leak allows air back into the pleural space during inspiration and results in a nonresolving pneumothorax

Incomplete seal of tissue around the chest tube insertion site (Fig. 10) can lead to backflow of atmospheric air into the pleural space due to the negative intrapleural pressure created during inspiration resulting in a persistent air leak. Capnography can be used to differentiate the source of air by measuring the CO_2_ level [28]. However, CT can also identify the unsuspected incomplete seal, which can be difficult to identify in the presence of extensive chest wall emphysema. CT can also confirm the location of the chest tube within the pleural space.

## Types of pleural fistula

There are two main types of pleural fistulas: the alveolopleural fistula (APF) and the bronchopleural fistula (BPF).

**Fig. 11:**
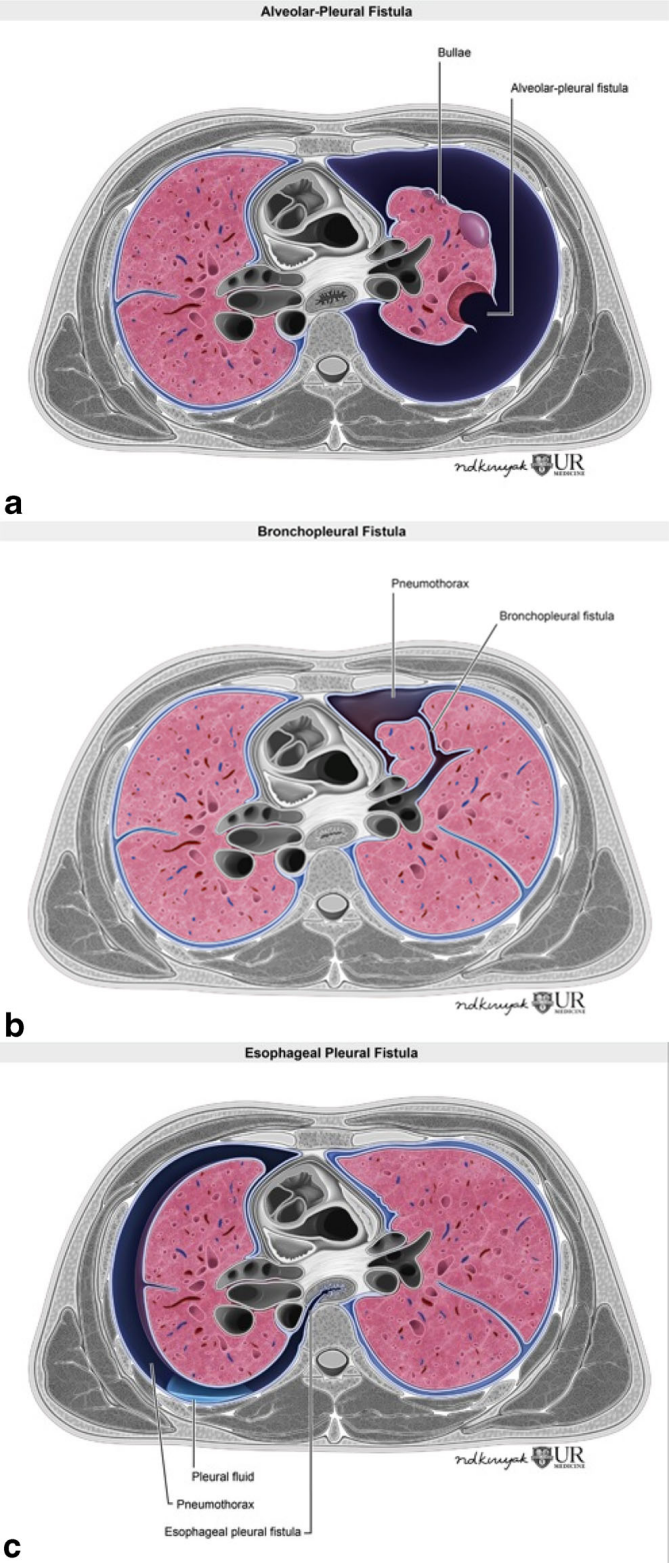
Illustrations demonstrating alveolopleural fistula (**a**), bronchopleural fistula (**b**), and the rare oesophageal pleural fistula (**c**). An alveolopleural fistula is characterized by communication between the pulmonary parenchyma distal to a segmental bronchus and the pleural space. A bronchopleural fistula denotes communication between the larger central airways such as the bronchi and the pleural space. Oesophageal pleural fistula signifies communication of the oesophagus with the pleural cavity. These fistulous communications often result in recurrent, persistent pneumothoraces

An alveolopleural fistula (APF) represents a communication between the pulmonary parenchyma distal to a segmental bronchus and the pleural space (Fig. 11a). Some also consider this as a peripheral bronchopleural fistula. This can be secondary to a ruptured bulla, cavitary neoplasm, necrotizing pneumonia, granulomatous infection/inflammation or post-thoracic intervention. A bronchopleural fistula (BPF) (Fig. 11b), on the other hand, is a communication between a main stem, lobar, or segmental bronchus and the pleural space [29]. In addition, a less common, third type of fistula may develop between the oesophagus and pleural space, referred to as an oesophageal pleural fistula (Fig. 11c).

## Causes of a persistent pneumothorax

### Ruptured bulla

**Fig. 12:**
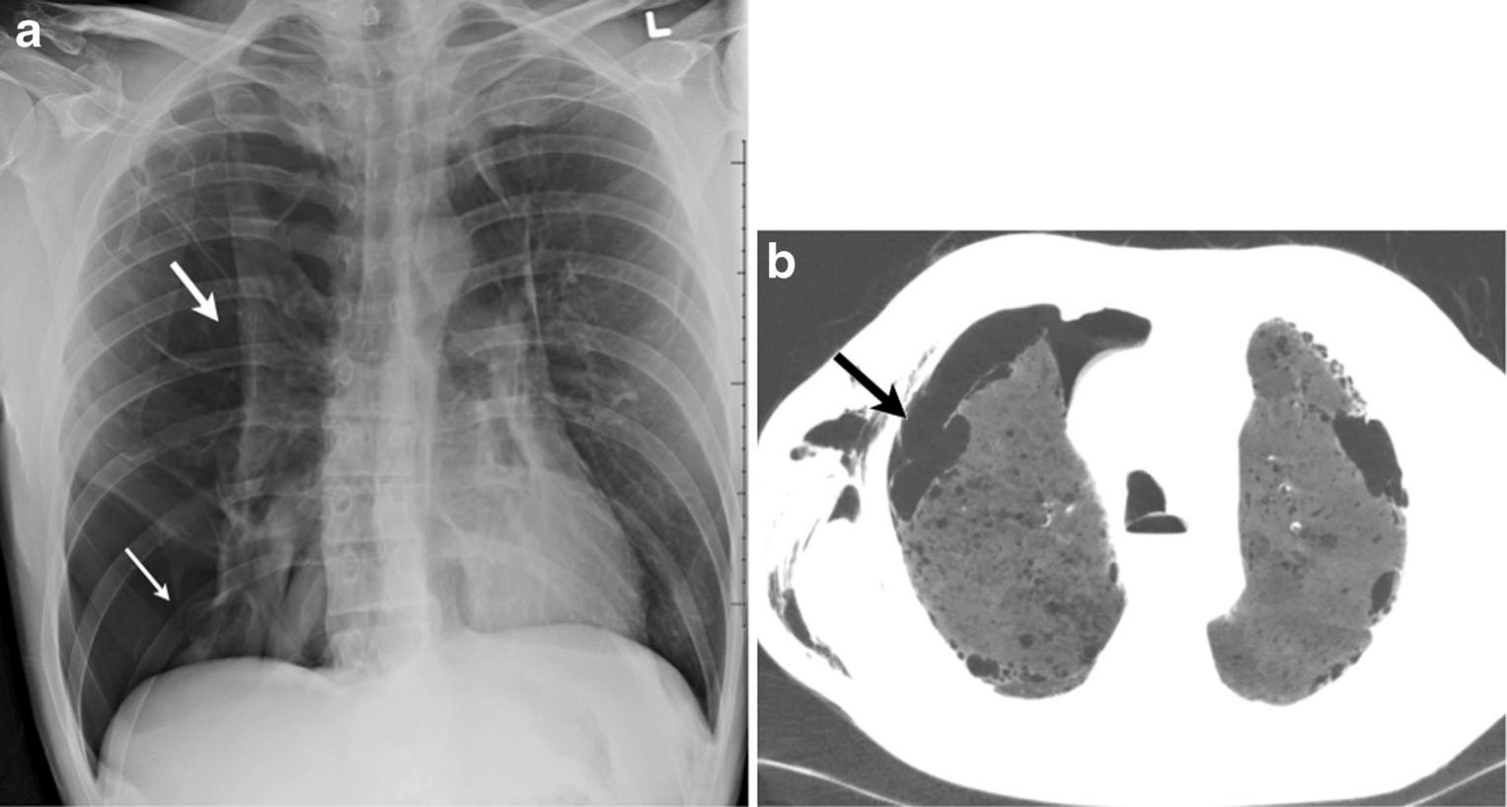
An 83-year-old smoker with chest pain and recurrent pneumothorax. Frontal chest radiograph on admission (**a**) demonstrates a large right pneumothorax (pleural interface marked by *white arrows*). Axial chest CT minimum intensity projection (minIP) image (**b**) demonstrates discontinuity of the walls of a bulla (*solid black arrow*) compatible with a ruptured bulla

Bullae and blebs are subpleural cystic gas containing spaces within the visceral pleura developing from enlargement of alveoli. These are distinguished based on size, a bleb being < 1 cm and a bulla > 1 cm. The wall of the bulla is < 1 mm in thickness [30]. Two different mechanisms have been postulated for their development. Congenital: due to rapid growth of upper lobe, which grows faster than the vasculature, or due to inherited genes such as HLA haplotype A2B40, alpha-1 antitrypsin phenotypes M1M2, and FBN1 genetic mutation. Acquired: negative intrapleural pressure is accentuated in taller patients or in those with emphysema. These are recognized on radiographs as round radiolucencies with thin walls. Blebs are easier to identify on CT. The sensitivity of high-resolution thin slice CT reconstruction is greater than routine thicker slice thickness reconstruction for the detection of bullae or blebs [31]. A ruptured bulla will demonstrate focal disruption or discontinuity of the wall leading to air leak into the pleural cavity (Fig. 12).

### Cystic lung diseases

When evaluating the lung parenchyma for aetiology of a persistent pneumothorax, it is important to evaluate for any underlying cystic lung diseases. The more common diffuse cystic lung diseases are: lymphangioleiomyomatosis and Langerhans cell histiocytosis (LCH) [32]. In addition, desquamative interstitial pneumonia (DIP), usual interstitial pneumonia (UIP), lymphocytic interstitial pneumonia (LIP), Birt-Hogg-Dube Syndrome, amyloidosis and metastasis can also cause cystic lungs, which can, in turn, cause pneumothorax.

**Fig. 13:**
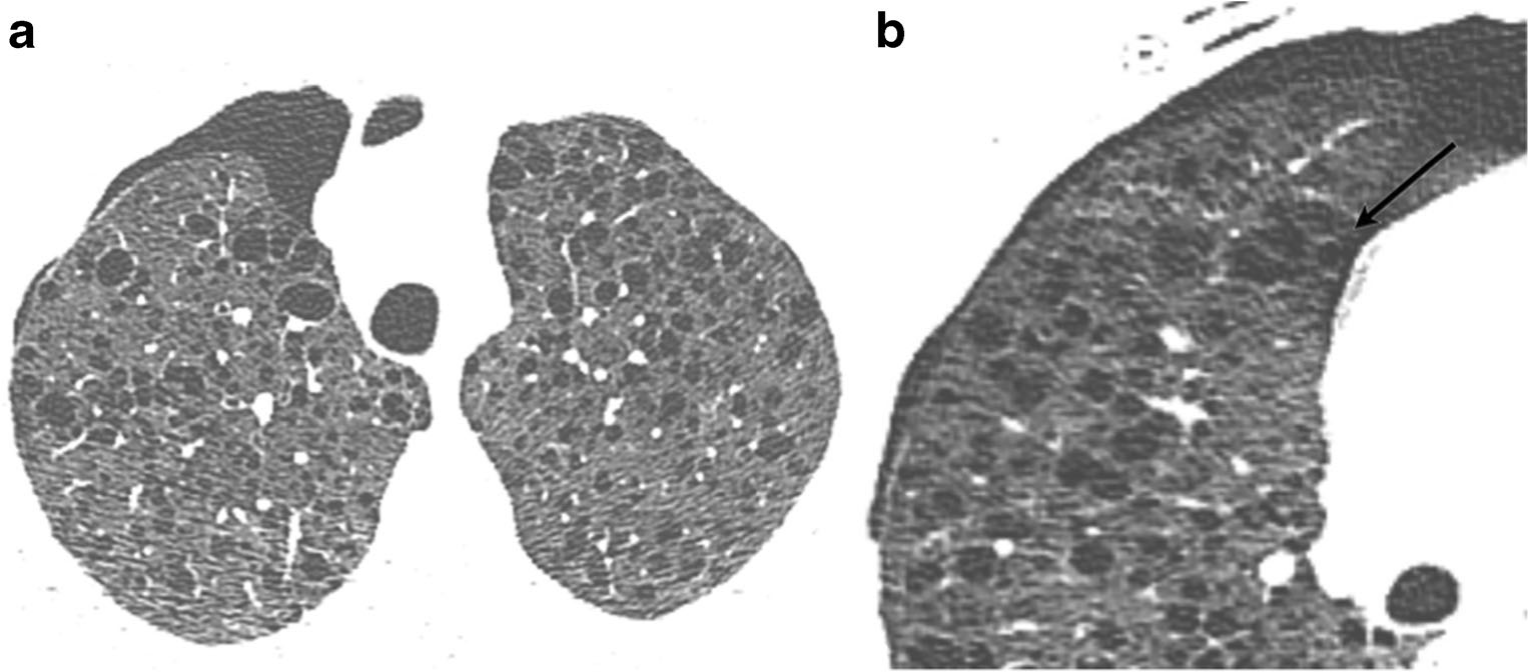
A 28-year-old patient with lymphangioleiomyomatosis, recurrent pneumothoraces and chest pain. Chest CT axial image (**a**) demonstrates multiple cysts in both lungs. Thin slice image (**b**) with a sharpened reconstruction kernel clearly demonstrates the discontinuity of the walls of a cyst (*arrow*) compatible with an alveolopleural fistula

Lymphangioleiomyomatosis (Fig. 13) is a systemic neoplasm that leads to smooth-muscle cell proliferation in the pulmonary interstitium. This is primarily seen in young women. It may be sporadic or associated with tuberous sclerosis complex (autosomal dominant). The cysts are thought to arise from air trapping resulting from peribronchiolar proliferation. Spontaneous or recurrent pneumothorax may be the presenting finding in up to 50 % of patients. On CT, these cysts are thin walled, round, and diffusely distributed in the central and peripheral parenchyma, with extension into the base [33]. Pulmonary LCH is seen in adults and is a smoking related lung disease characterized by peribronchiolar infiltration of inflammatory cells, forming nodules which cavitate, resulting in thin and thick walled cysts, sometimes with bizarre shapes and with sparing of the costodiaphragmatic sulci. Subpleural nodules and interstitial thickening are also present.

### Pulmonary infections/abscess

Necrotizing pneumonia and pulmonary abscesses, though uncommon, are associated with very high mortality [34]. Patients with altered consciousness carry an increased risk for aspiration, and are therefore particularly predisposed to developing lung infections. This subgroup includes alcoholics, patients with proximal lung cancer, diabetes, seizures or cerebrovascular diseases and those with compromised immunity [35, 36].

**Fig. 14:**
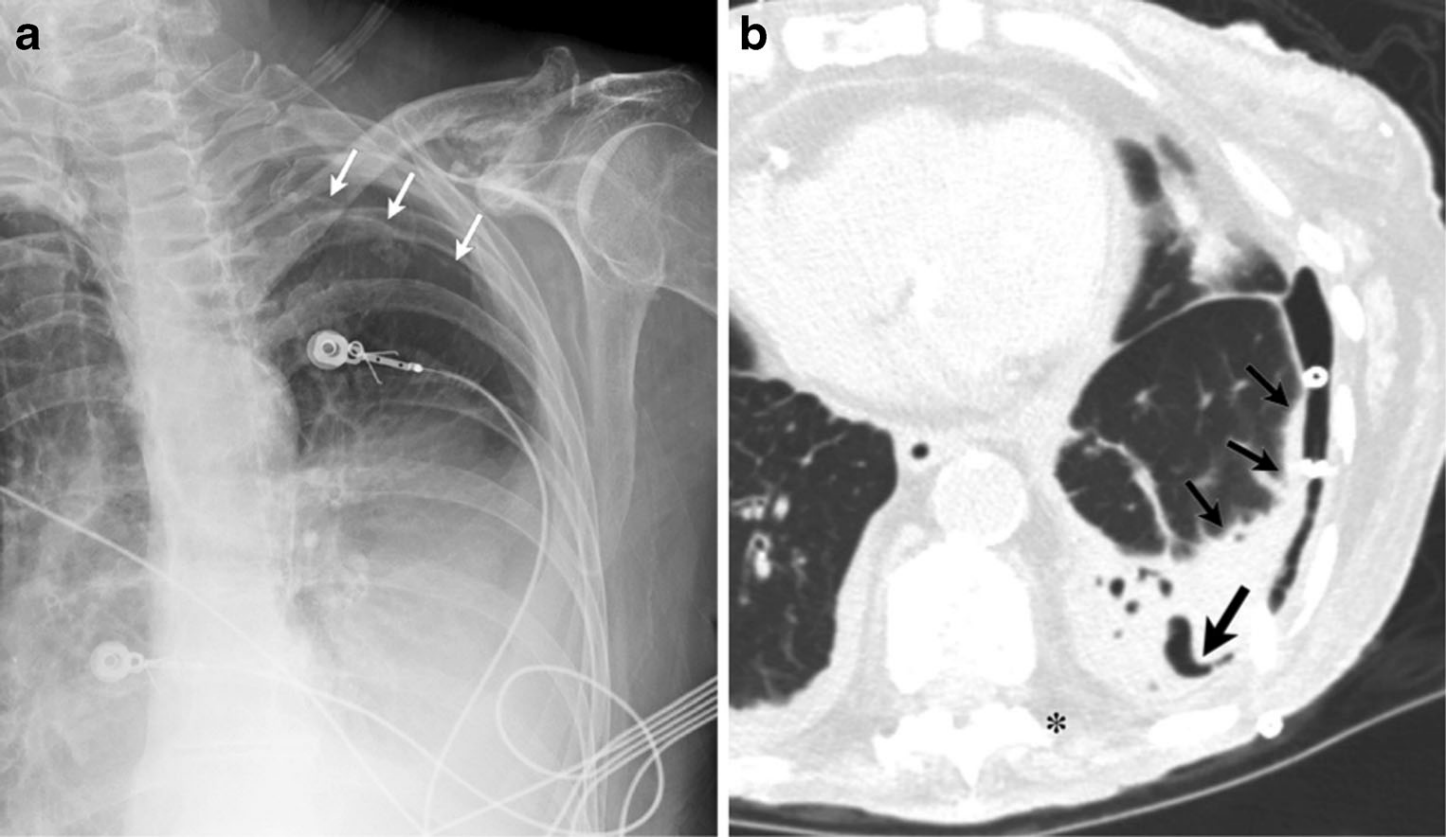
A 70-year-old female with a history of left-sided empyema, status post drainage. Frontal chest radiograph (**a**) demonstrates a small left apical pneumothorax (*white arrows*). Axial CT (**b**) demonstrates a broncho-pleural fistula (*thicker black arrow*) secondary to parenchymal necrosis. In addition, pleural thickening (fibrothorax) surrounding the left lower lobe (*small arrows*) “traps” the lung, preventing it from fully expanding. This is also an example of an ex vacuo pneumothorax

The two most common complications of these infectious parenchymal necroses are empyema and persistent air leak from a BPF or APF (Fig 14). The adjacent lower lung may get entrapped from this active pleural disease. It has been suggested that the presence of pneumothorax in a patient with pneumonia should raise suspicion for necrotizing pneumonia [37]. Pulmonary tuberculosis caused by Mycobacterium, and non-mycobacterial infections can also cause APF or BPF and pneumothorax [38, 39].

### Parasitic pleural diseases

APF with resultant pneumothorax can also be seen with amebiasis, echinococcosis and paragonimiasis [40].

### Cancer and pneumothorax

**Fig. 15:**
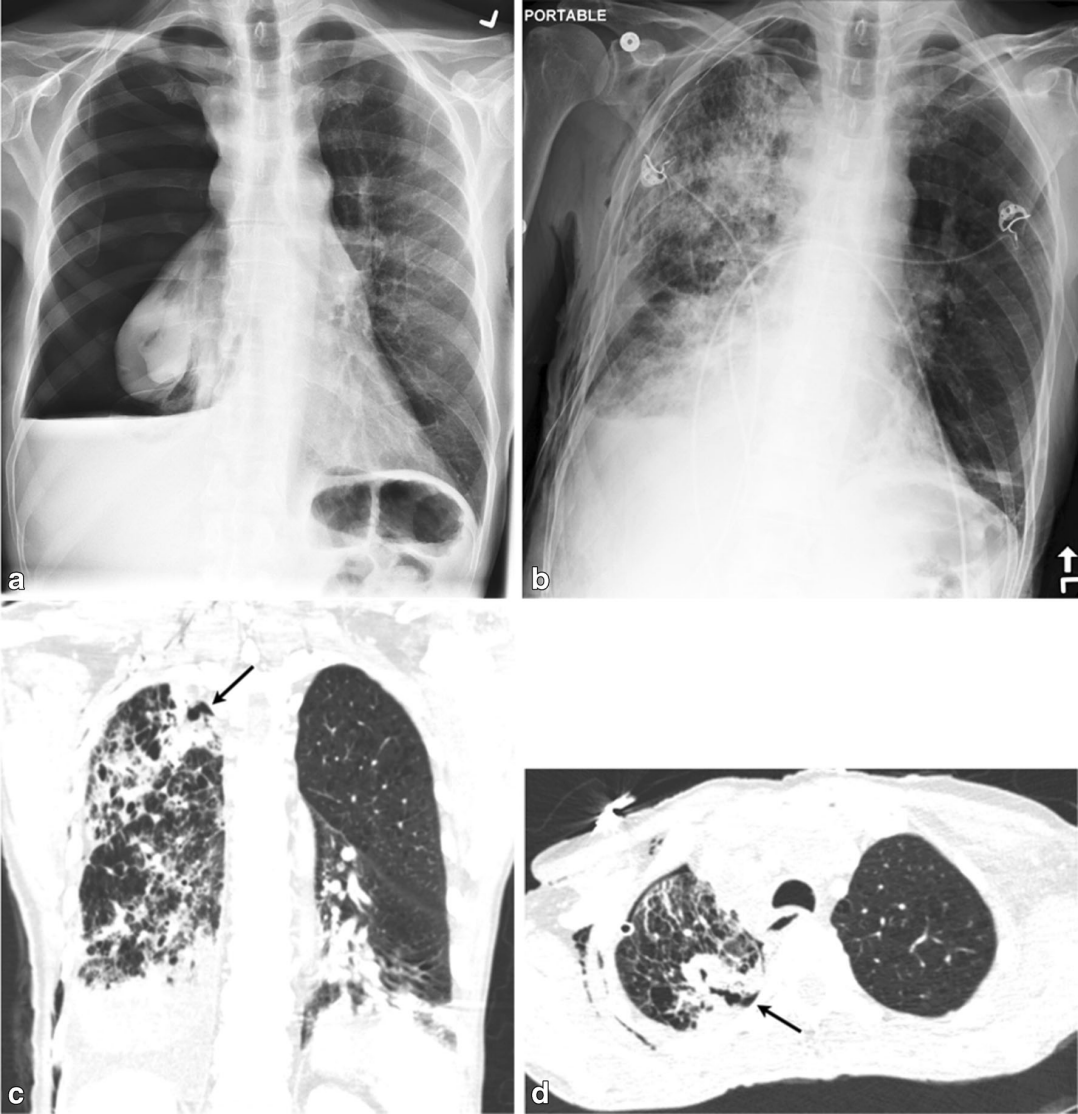
A 56-year-old male presented to the ED with right-sided chest pain. Frontal chest radiograph (**a**) demonstrated a large right hydro pneumothorax, which did not completely resolve after chest tube placement (**b**). Coronal (**c**) and axial (**d**) CT images demonstrated a cavitating cancer within the right upper lung with an associated alveolopleural fistula (*arrow*). In addition, right paratracheal lymphadenopathy is seen. Extensive alveolar opacities in the re-expanded lung are consistent with pneumonia and reexpansion edema

**Fig. 16:**
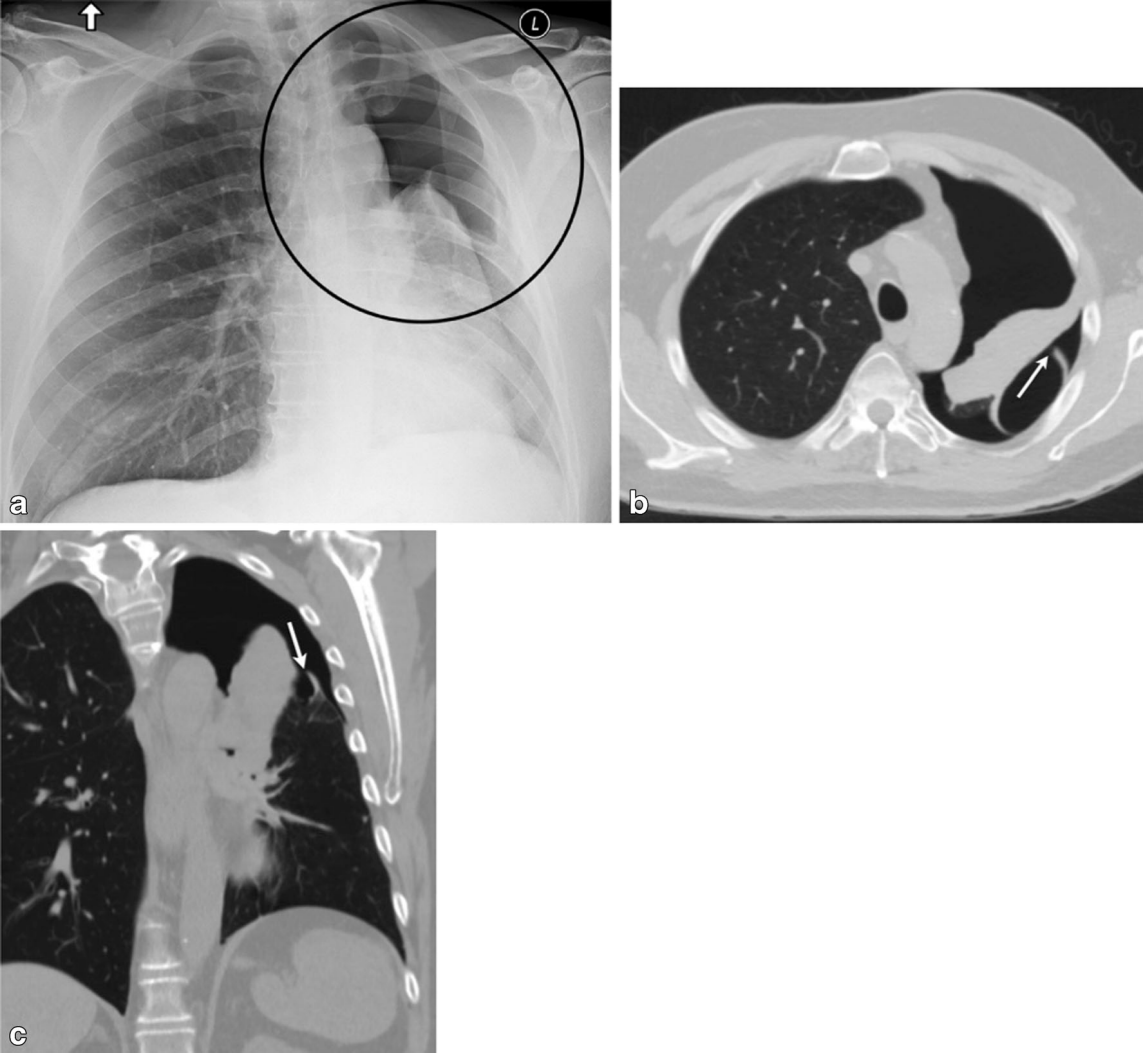
A 60-year-old male with left upper lung carcinoma; post radiation therapy. Frontal chest radiograph (**a**) demonstrates a left apical pneumothorax (*circle*). Axial (**b**) and coronal (**c**) CT images demonstrate discontinuity of visceral pleura (*arrow*) with associated alveolopleural fistula

Rarely, spontaneous pneumothorax can be the initial manifestation of an underlying lung cancer. In such cases, pneumothorax can arise secondary to rupture of the necrotic neoplastic tissue into the pleural cavity (Fig. 15, a-d), rupture of a subpleural bleb or formation of interstitial air due to partial bronchial obstruction by the tumour, complication of radiation therapy (Fig 16, a–c) or chemotherapy [41].

### Lung metastases

Lung metastases from sarcoma can result in pneumothorax; which, though rare, carry a high mortality [42]. Metastases from mesenchymal sarcomas can also present with cystic lung disease, which can be complicated by pneumothorax. Necrosis of a peripheral metastasis post chemotherapy (doxorubicin in particular) or radiation can also cause pneumothorax. The most common cell types causing a pneumothorax are: osteosarcoma, angiosarcoma and Ewing’s sarcoma. Presence of a pneumothorax is a poor prognostic indicator in these patients, and most of these pneumothoraces require treatment. Increasingly, microwave or radiofrequency ablation is being used to treat lung cancer in patients who are not operative candidates; pneumothorax is a major complication of these procedures [43, 44].

### Post surgical

BPF is one of the most morbid postoperative complications after a lobectomy or pneumenectomy [45]. Incidence is higher after pneumenectomy (2–20 %) compared to lobectomy (0.5–3 %) [45]. Surgical management is required to limit airflow across the fistula, close fistula and evacuate pleural space; protecting normal lung from spillage of pleural fluid is equally important.

**Fig. 17:**
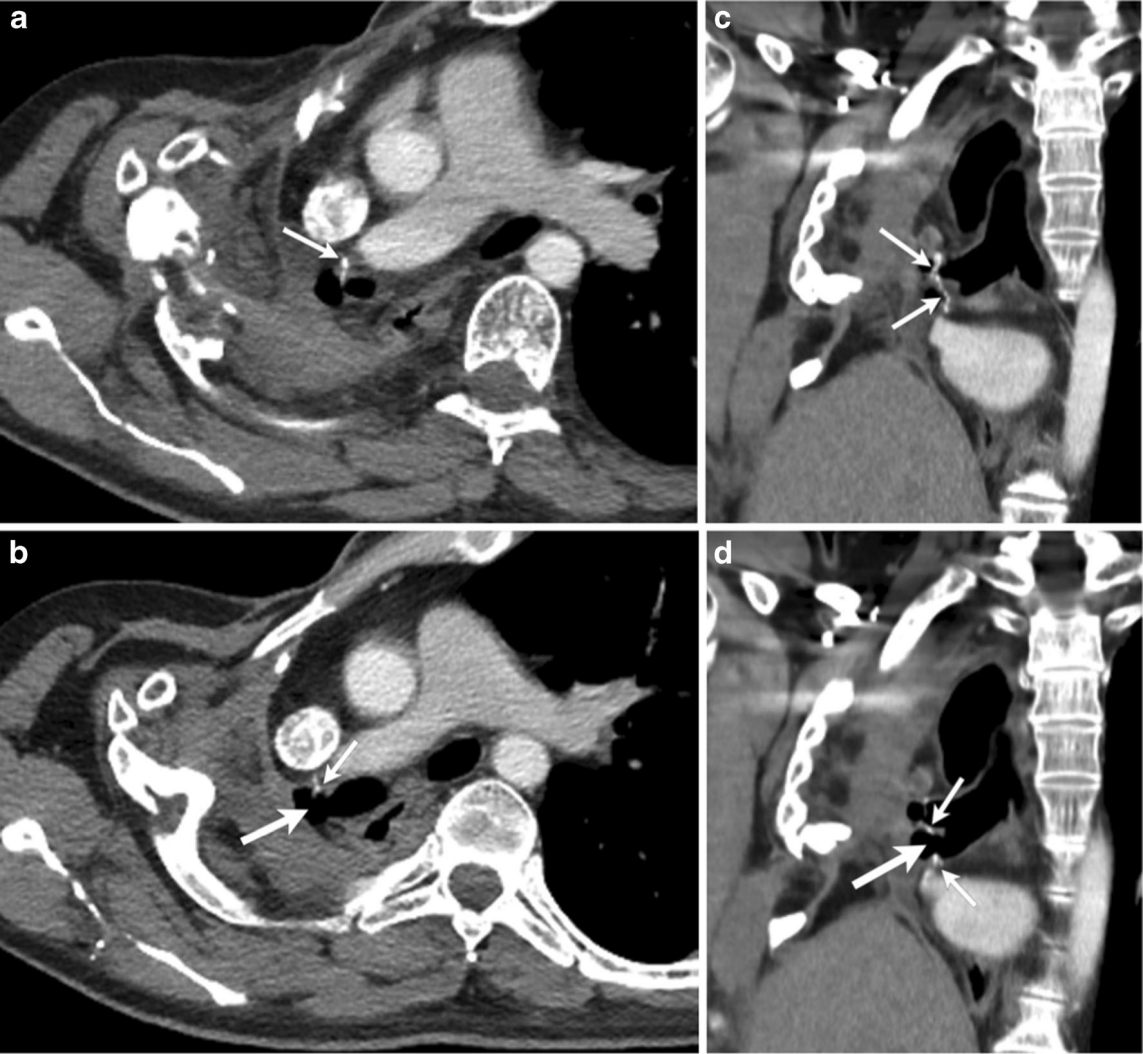
34-year-old male with history of gunshot injury; presenting with persistent pneumothorax post pneumonectomy. Axial (**a**,**b**) and coronal (**c**,**d**) post pneumonectomy CT images demonstrate a dehiscent bronchial stump communicating with the pleural space (*thick arrow*). Notice discontinuity of the suture line (*small arrows*)

CT (Fig. 17) is an important noninvasive tool to identify the location and size of this defect, and is considered superior to bronchoscopy [46]. On radiographs, a new or an increased pneumothorax, decrease in the air fluid level, lack of progressive fluid accumulation or a shift of the mediastinum away from the resected side after a pneumonectomy, is suggestive of BPF. On CT, extraluminal air bubbles adjacent to a stump may be identified (Fig. 17). After surgery, documenting complete resolution of pneumothorax is important. From the perspective of air travel, the current recommendation is to delay air travel by 1–3 weeks post surgery or post resolution of pneumothorax [47].

### Penetrating injury

**Fig. 18:**
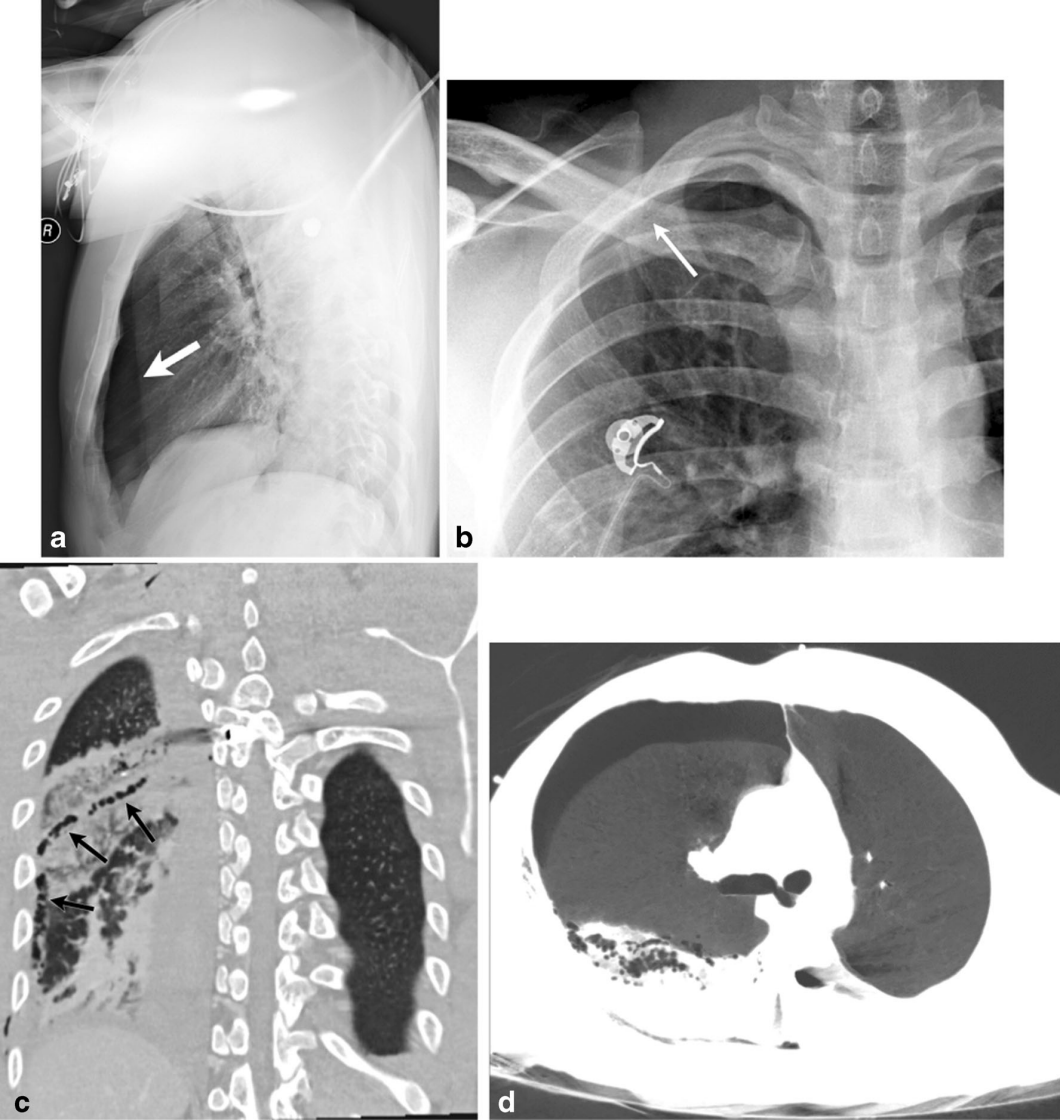
A 29-year-old male, status post gunshot wound. Emergently performed supine lateral chest radiograph at initial presentation (**a**) demonstrated an anterior right pneumothorax (*solid arrow*). Follow-up frontal chest radiograph (**b**) obtained 7 days later revealed a persistent right apical pneumothorax (*small white arrow*). Upon retrospective review, an alveolar- pleural fistula (*black arrows*) was present on this coronal reformatted image from the initial CT evaluation (**c**). This was secondary to lung laceration sustained along the bullet track. Axial minIP (**d**) image demonstrates the communication of this pulmonary laceration with the pneumothorax

**Fig. 19:**
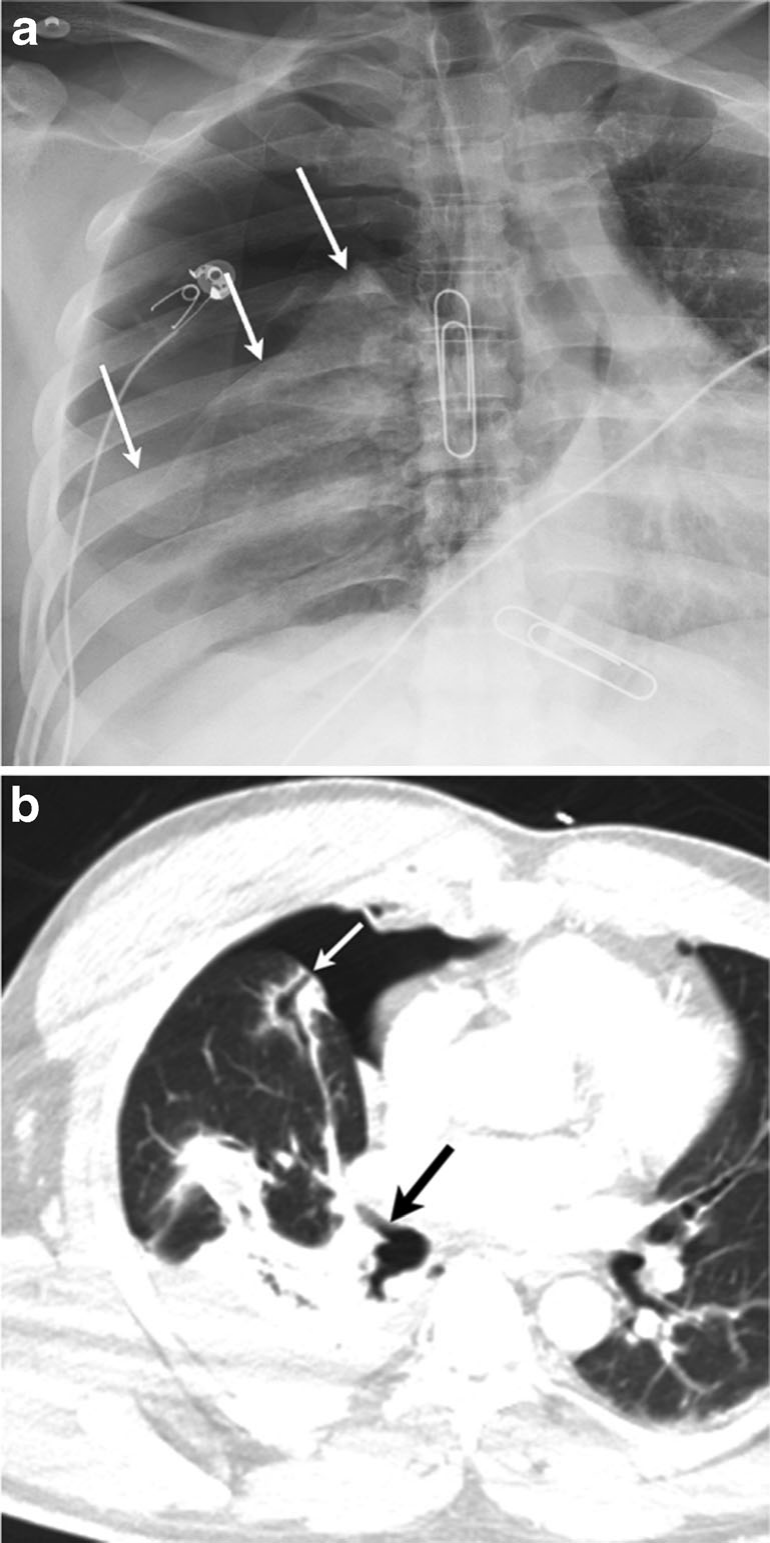
A 47-year-old male presented to the ED with a stab wound to the right chest. Frontal chest radiograph (**a**) demonstrates a right-sided tension pneumothorax (*arrows*). Axial image from CT (**b**) performed the same day demonstrated direct communication of the right middle lobe bronchus (*large arrow*) with the pleural space (*small arrow*)—a bronchopleural fistula

Thoracic penetration injury may be due to ballistic trauma such as gun shot wounds (Fig. 18), or due to nonballistic trauma such as stab wounds (Fig. 18) or rib fractures (Fig. 19). These can cause either a BPF or an APF.

With ballistic injury, direct tissue laceration along the trajectory of the bullet forms a permanent cavity, followed by a temporary cavity due to pressure gradients radial to the trajectory of the bullet [48]. The temporary cavity depends on the velocity and size of the bullet [49]. The penetrating projectile disrupts the chest wall, parietal pleura, visceral pleura, and alveolar wall. This results in a direct communication of the atmospheric air with the pleural space or/and alveolus with the pleura space.

**Fig. 20:**
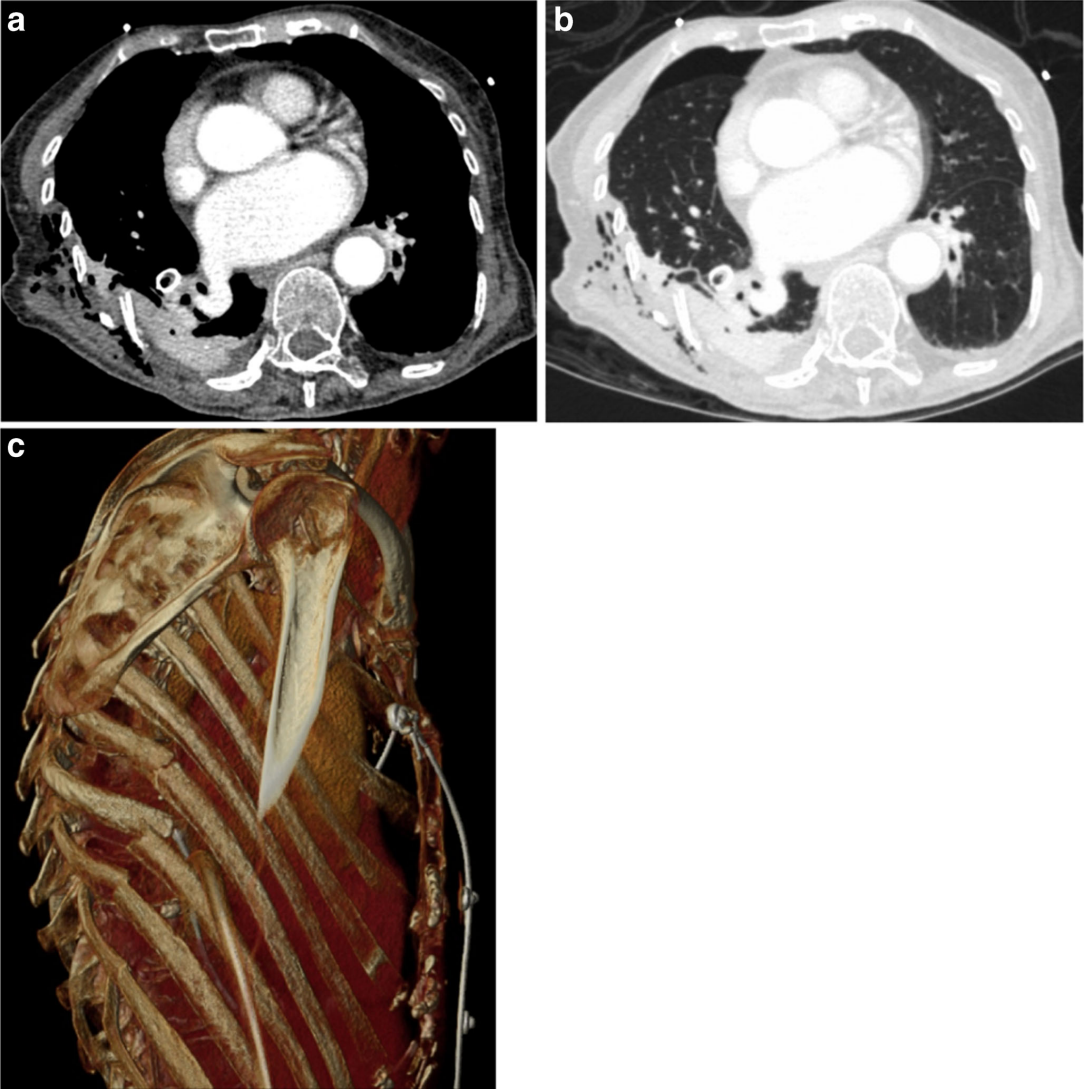
A 40-year-old male presented to the ED with multiple rib fractures sustained after a fall from height. Mediastinal window image from axial chest CT (**a**) demonstrated a markedly displaced right-sided posterior rib fracture. The same image on lung window (**b**) demonstrated a small right pneumothorax secondary to bronchopleural fistula secondary to traumatic lung laceration. Right posterior chest wall soft tissue emphysema was seen on both (**a**) and (**b**). VR image (**c**) demonstrates the displaced rib fractures

Similarly, unrecognized stab wounds (Fig. 20) can cause communication of the atmospheric air with the pleural space, and unless recognized and closed, will lead to a persistent pneumothorax. Displaced fractured ribs in blunt thoracic trauma can cause penetrating lung injury [50]. This can result in an APF or a pulmonary laceration. Pulmonary laceration is different from solid organ laceration such as liver or spleen. Due to the elastic recoil of the lungs, normal tissue surrounding the laceration recoils to form oval or round defects that can lead to formation of BPF [51]. Laceration associated with rib fracture is described as a Type 3 (rib penetration tear), which is a small peripheral defect associated with pneumothorax.

### Others

**Fig. 21:**
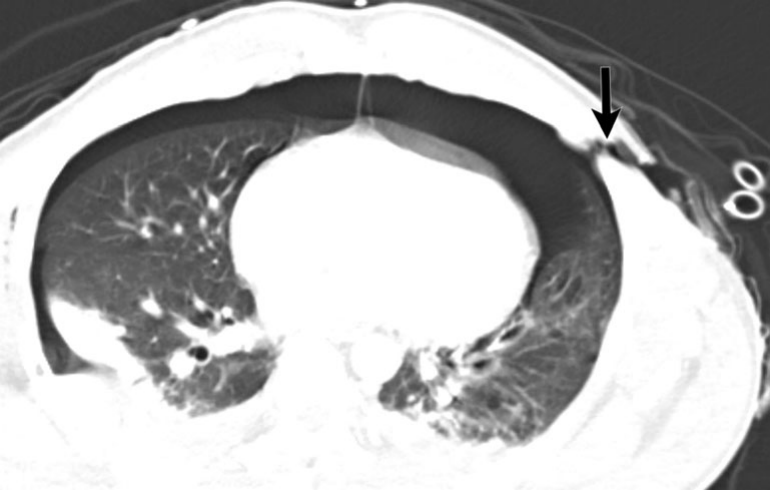
A 32-year-old female presented with a stab wound to the left anterolateral chest. Lung window axial CT image through the mid-chest demonstrated an irregular, gas-filled defect along the left anterior chest wall extending to the pleural cavity (*arrow*). This allowed for direct communication of the pleural space with atmospheric air. Associated large left and moderate right pneumothoraces were present. Pneumothorax is likely to persist in this scenario of an open wound communicating with the atmosphere

**Fig. 22:**
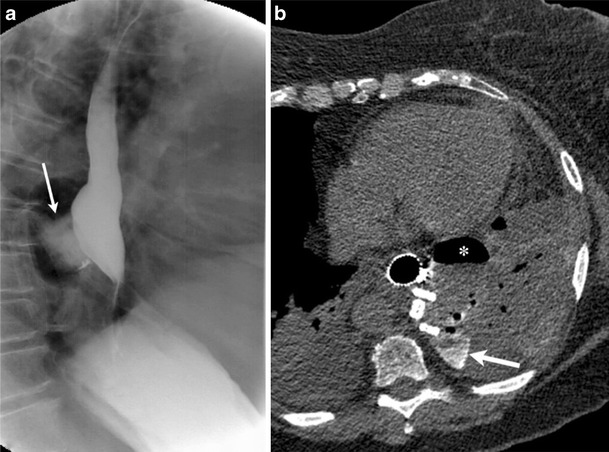
A 63-year-old male with oesophageal injury and resultant oesophageal-pleural fistula; post-Nissen fundoplication. Lateral image from contrast oesophagram using Omnipaque-350 (**a**) demonstrated extraluminal leak and subsequent mediastinal pooling (*arrow*) of orally administered contrast. Axial CT (**b**) demonstrates interval oesophageal stent placement. A complex left-sided paramediastinal fluid collection is seen containing both contrast (*arrow*) and air (*)

Esophagorespiratory fistulas (Fig. 21) can be due to oesophageal or lung cancer [52]. These can be esophagotracheal, esophagobronchial or an esophagopulmonary fistulae. Esophagopleural and gastropleural fistulas (Fig. 22) are rare and can occur as a complication of thoracic surgery, oesophageal disease or cancer [53], and often present with empyema thoracis.

## Treatment

**Fig. 23:**
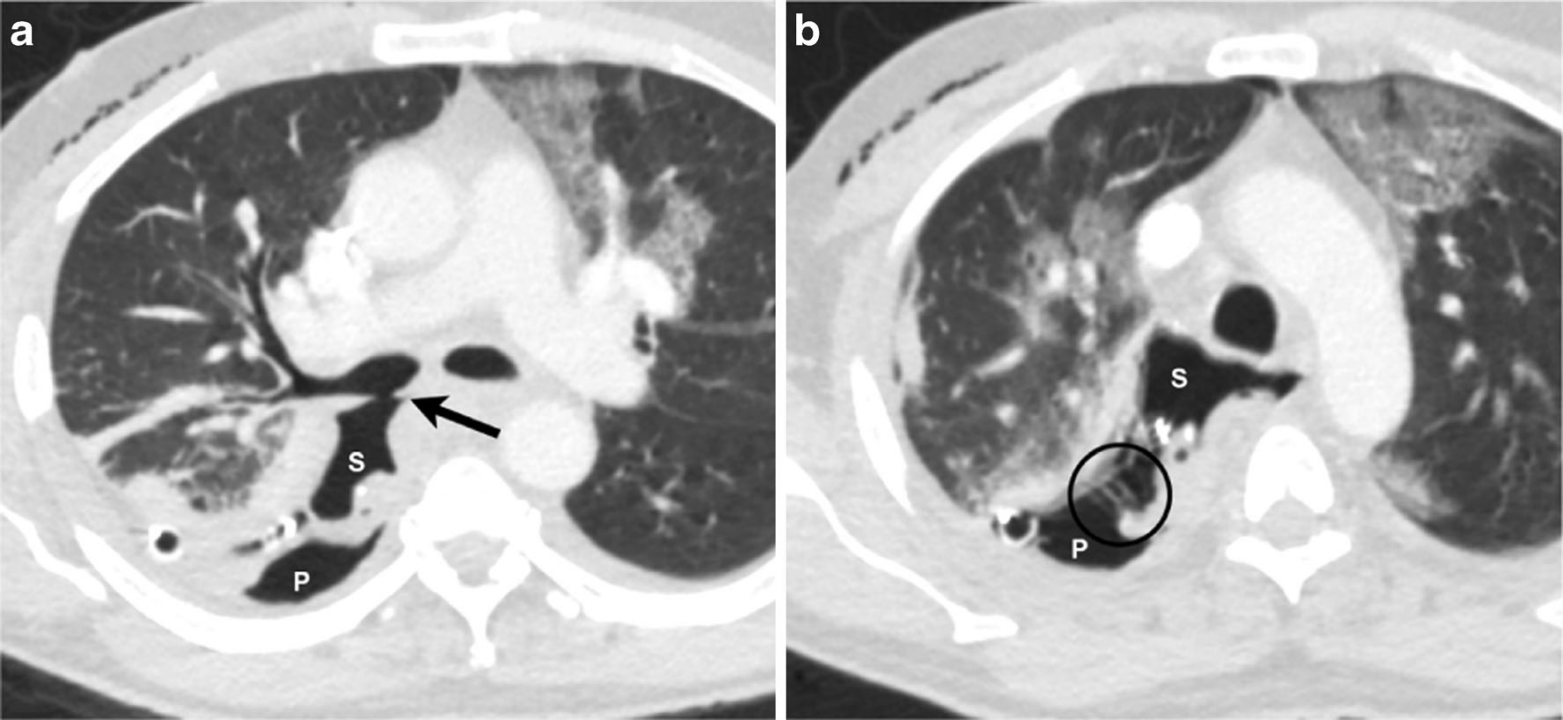
A 47-year-old male, post gastric pull through for oesophageal cancer. Axial CT (**a**) image through the mid-chest demonstrates direct communication of the right main stem bronchus with the stomach (S) (*black arrow*) resulting in a broncho-gastric fistula. Several slices inferiorly (**b**), posterior gastric wall is dehiscent (*circle*) and communicates with the pleural space (P), resulting in a gastropleural fistula. Note the chest tube within the posterior right pleural space

**Fig. 24:**
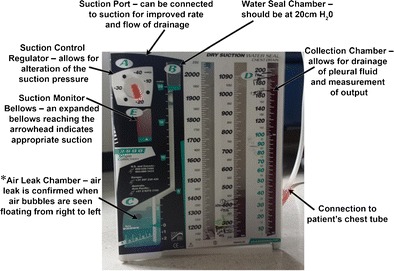
Water seal drainage system. Bubbling within the air leak chamber (*) should cease within 24 h. If an air leak has already sealed, bubbling will be seen only with cough or Valsalva. Continuous bubbling indicates a large air leak. A cardinal sign of a blocked thoracic tube is failure of the fluid column within the tube to fluctuate with coughing or respiration

Pneumothorax can be treated using a conservative approach, needle aspiration, chest drain, suction (Fig. 23) or surgery [54]. Ambulatory treatment is, however, not recommended for a persistent pneumothorax; often, these patients require admission and continued observation. Surgical intervention may be needed for persistent air leak beyond 4 days. Tube thoracotomy remains the mainstay for treating pneumothorax (small bore < 14 F, large bore > 14 F). Increasingly, CT guided intercostal pleural catheter placement is being used to treat loculated pneumothorax (Fig. 24).

Blebectomy with pleurectomy: Surgical resection of bullae is indicated in patients with second episode of spontaneous pneumothorax. It can also be performed in patients who have first episode of spontaneous pneumothorax with a prolonged air leak (greater than 72 h), incomplete expansion of the lung, bilateral pneumothoraces, hemothorax, or tension pneumothorax. In addition, pleura is resected posteriorly, anteriorly and laterally. Mediastinal and diaphragmatic pleural surfaces are abraded to remove pleura. Surgical pleurectomy has the lowest rate of pneumothorax recurrence (1 %), but is associated with increased pain and longer hospital stays. VATs pleurectomy has a slightly higher rate of pneumothorax reoccurrence (5 %), but is associated with lower morbidity and shorter hospital stays. Chemical pleurodesis has historically been done with talc. It is currently much less preferred than surgical pleurectomy.

Intrabronchial valves are umbrella-shaped devices (5–7 mm diameter) that limit airflow to distal lung and can be placed using bronchoscopic guidance [55]. These valves have been approved through the Humanitarian Device Exemption for persistent air leaks after segmentectomy, lobectomy, and lung volume reduction surgery and as “off-label” use for APF.

Esophagorespiratory fistulae are life threatening and require urgent treatment. CT is important to define the anatomy of the airway and oesophagus. It helps identify the defect and define the optimal landmarks for stent placement. Surgical management includes gastric bypass. Feeding gastrostomy or jejunostomy may be used for palliation.

Novel methods proposed to reduce persistent pneumothorax include blood patch pleurodesis [56] and portable thoracic suction drainage systems [57]. To reduce the incidence of postoperative air leak, the use of free pericardial fat pad [58], and of human fibrinogen-thrombin patch [59] have also been proposed.

## Conclusion

In conclusion, persistent pneumothorax is a diagnostic conundrum. Specific aetiology may be hidden in plain sight. Careful evaluation of radiographs and CT may identify the cause, leading to early diagnosis and treatment.
